# Parenchymal and Dyshoric Fibrillar Amyloid Pathology in the rTg-D Rat Model of Cerebral Amyloid Angiopathy Type-2

**DOI:** 10.1016/j.ajpath.2025.06.008

**Published:** 2025-07-17

**Authors:** Joseph M. Schrader, Feng Xu, Xiaoyue Zhu, Mark Majchrzak, Judianne Davis, William E. Van Nostrand

**Affiliations:** ∗ George & Anne Ryan Institute for Neuroscience, University of Rhode Island, Kingston, Rhode Island; † Department of Biomedical and Pharmaceutical Sciences, University of Rhode Island, Kingston, Rhode Island

## Abstract

Cerebral amyloid angiopathy (CAA) is a common age-related disorder, a prominent comorbidity of Alzheimer disease (AD), and causes vascular cognitive impairment and dementia. A previously developed novel transgenic rat model (rTg-D) expresses the human familial CAA Dutch E22Q mutant amyloid β-protein in brain with hemizygous (HEM) animals developing arteriolar CAA type-2 pathology. In this study, homozygous (HOM) rTg-D rats developed more extensive CAA type-2, characterized by abundant fibrillar amyloid accumulation, including parenchymal congophilic plaques and dyshoric vascular amyloid. Similar to the vascular amyloid, fibrillar amyloid plaques in rTg-D HOM rats were predominantly composed of amyloid β40. The rTg-D HOM rats exhibited pronounced astrocytic and microglial responses as well as phosphorylated tau accumulating in surrounding dystrophic neurites and early tangle-like structures. Cerebral proteomic analyses revealed that while rTg-D HEM rats and rTg-D HOM rats shared some common differentially expressed proteins compared with wild-type rats, rTg-D HOM rats exhibited many more elevated proteins. Because the parenchymal fibrillar plaques of rTg-D HOM rats resemble those seen in AD, the cerebral proteomes were compared between rTg-D HOM rats and a transgenic rat model of AD. This analysis showed that they shared many differentially expressed proteins and activated pathways, including activation of transforming growth factor-β1 signaling and swarming of neutrophils. In conclusion, the present findings show that rTg-D HOM rats develop more severe CAA type-2 pathology than rTg-D HEM rats coupled with AD-like pathologic features, making them a valuable model for studying the intersection of vascular amyloidosis and neurodegeneration.

## Graphical Abstract

**Figure:**
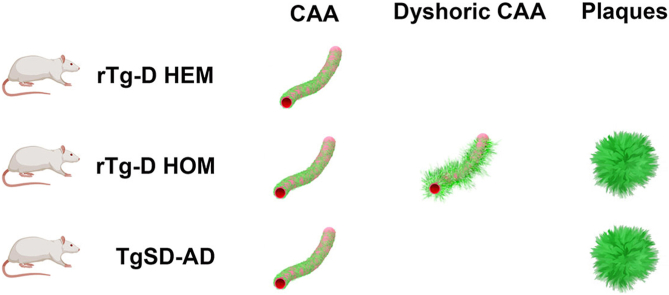

Cerebral amyloid angiopathy (CAA) is a significant and common form of cerebral small-vessel disease, particularly in the elderly population, characterized by the deposition of fibrillar amyloid-β (Aβ) in the walls of blood vessels in the brain. This leads to significant vascular and neurodegenerative complications.^1^, ^2^, ^3^ CAA is present in over half of individuals aged >80 years, and is common in patients with Alzheimer disease and related disorders (ADRD).^2^^,^^4^, ^5^, ^6^ The deposition of amyloid β in the blood vessel walls can compromise vessel integrity, leading to various pathologic outcomes, including perivascular neuroinflammation, impaired brain waste clearance, cerebral infarction, microbleeds, and, in severe cases, intracerebral hemorrhages.^2^^,^^5^^,^^7^, ^8^, ^9^, ^10^, ^11^, ^12^, ^13^ Through these mechanisms, CAA contributes to vascular-mediated cognitive impairment and dementia, worsening the cognitive decline in patients with ADRD.^4^, ^5^, ^6^^,^^14^ Two prominent forms of CAA exist: CAA type-2 and CAA type-1.^15^ CAA type-2, the more common form, is characterized by amyloid accumulation in the walls of meningeal and cortical small arteries and arterioles.^1^^,^^2^^,^^15^ It is often associated with aging and is prevalent in older individuals with ADRD. In contrast, the less common CAA type-1 amyloid primarily deposits along capillaries and is strongly associated with the apolipoprotein ε4 allele, a known genetic risk factor for Alzheimer disease (AD).^2^^,^^15^^,^^16^ CAA type-1 can also coexist with CAA type-2, leading to more complex vascular and neurologic manifestations.

In addition to the prominent CAA found in ADRD and in sporadic cases of this condition, several hereditary forms of CAA have been identified that result from specific mutations that generally reside within the middle of the Aβ region of the amyloid β precursor protein (*AβPP*) gene.^17^, ^18^, ^19^, ^20^ The first recognized form is the Dutch-type CAA, which results from an E22Q substitution in Aβ and causes early-onset and severe cerebral vascular amyloid deposition. Notably, Dutch-type CAA is characterized by the absence of parenchymal fibrillar plaques and phosphorylated tau tangles commonly seen in AD.^17^^,^^18^^,^^21^, ^22^, ^23^ The E22Q mutation markedly enhances the fibrillogenic and pathogenic properties of Aβ compared with the wild-type peptide, nonmutated Aβ^24^^,^^25^ and can produce amyloid fibrils with a distinct structure.^26^ Clinically, patients with Dutch-type CAA present with recurrent, often fatal, intracerebral hemorrhages accompanied by progressive vascular-mediated cognitive impairment and dementia.^23^, ^24^, ^25^^,^^27^

The rTg-D rat model of CAA type-2 produces Dutch E22Q CAA mutant Aβ peptides in brain and develops key pathologic features of human disease, including age-related arteriolar amyloid deposition, loss of vascular smooth muscle, cerebral microhemorrhages, small-vessel occlusions, and cognitive impairments.^28^^,^^29^ The rTg-D rats, maintained in a hemizygous (HEM) state, exhibit some variability in their age of onset and severity of CAA. In the present study, rTg-D rats were bred to homozygosity (HOM) in an attempt to accelerate CAA formation while producing more uniform pathology. rTg-D HOM rats not only developed more extensive CAA, but also unexpectedly exhibited abundant fibrillar parenchymal amyloid plaques and dyshoric vascular amyloid, where vascular amyloid fibrils protrude into the adjacent parenchyma. These pathologic features triggered robust glial responses, phosphorylated tau pathology, and widespread changes to the cerebral proteome more reminiscent of AD pathology.

## Materials and Methods

### Animals

All work with animals was approved by the Institutional Animal Care and Use Committee at the University of Rhode Island (project number AN1718-008) and performed in accordance with the US Public Health Service's Policy on Humane Care and Use of Laboratory Animals and in compliance with the Animal Research: Reporting of *In* *Vivo* Experiments (ARRIVE) guidelines.^30^ rTg-D rats expressing human AβPP harboring the Swedish K670N/M671L mutations and Dutch E693Q CAA mutation in neurons under control of the murine Thy1.2 promoter were generated, genotyped, and characterized, as previously described.^28^ The rTg-D rats used in the present study were maintained either as HEM or HOM for the transgene and maintained on Sprague-Dawley (SD) background.

For analysis of a transgenic rat model of AD pathology, TgF344-AD rats^31^ were imported from the Rat Resource and Research Center [strain: F344-Tg(Prp-APP,Prp-PS1)19/Rrrc; Columbia, MO]. The TgF344-AD rat line expresses familial Alzheimer disease (FAD) mutant human *AβPP* (AβPPsw) and FAD mutant presenilin 1 (PS1ΔE9) transgenes, both under transcriptional control of a mouse prion promoter. The imported TgF344-AD rats, on a Fisher 344 background, were subsequently bred onto a SD background for 10 generations (hence designated TgSD-AD).^32^ This was performed so that both the TgSD-AD rat model and rTg-D CAA rat model (HEM and HOM) were all on the same SD background to facilitate cross-line comparisons. Non-transgenic, SD rats served as wild-type (WT) controls. All rats were housed in a controlled room (22°C ± 2°C and 40% to 60% humidity) on a standard 12-hour light cycle. Rat chow and water were available *ad libitum*. Depending on the measure conducted, sample sizes for all groups ranged between *n* = 5 and *n* = 12. No notable sex differences in pathology have been observed in rTg-D HEM and HOM rats. However, both female and male rTg-D HEM, rTg-D HOM, and WT rats were included in all experimental groups, striving for an even balance between sexes in the experimental groups as possible, although the study was not sufficiently powered to conclusively report on sex differences within these models. In regard to TgSD-AD rats, only males were available for the present study, but again no notable sex differences in pathology have been observed or reported.^31^

### Brain Tissue Collection and Preparation

Euthanized rats were perfused with cold phosphate-buffered saline, and forebrains were removed and dissected through the midsagittal plane. One hemisphere was fixed with 4% paraformaldehyde overnight at 4°C and subjected to increasing concentrations (10%, 20%, and 30%) of sucrose in phosphate-buffered saline, then embedded in OCT compound (Sakura Finetek Inc., Torrance, CA) and snap frozen in dry ice. Sagittal sections were cut at 10 μm thickness using a Leica CM1900 cryostat (Leica Microsystems Inc., Bannockburn, IL), placed in a flotation water bath at 40°C, and then mounted on Colorfrost/Plus slides (ThermoFisher Scientific, Houston, TX). For proteomic analyses, collected hemispheres were frozen on dry ice and stored at –80°C before lysis for proteomic analysis. For laser capture microdissection, sagittal sections were prepared from hemispheres of fresh frozen brain tissue on laser microdissection framed membrane slides (Leica, Buffalo Grove, IL). Before laser capture microdissection, sections were prestained for amyloid using thioflavin S, allowing for specific isolation of amyloid plaques, and microvessel presenting with vascular amyloid deposits. Laser capture microdissection was performed using a Leica laser capture microdissecting microscope (Leica, Buffalo Grove, IL). Three hundred parenchymal plaque and vascular amyloid deposits were collected from each animal for subsequent enzyme-linked immunosorbent assay (ELISA) analysis.

### Immunoblot Analysis of Human AβPP

Forebrain tissues from approximately 12-month–old rTg-D HET and HOM rats were homogenized in 10 volumes of 50 mmol/L Tris-HCl (pH 7.5) containing 150 mmol/L NaCl, 1% SDS, 0.5% Nonidet P-40, 5 mmol/L EDTA, and proteinase inhibitor cocktail (Roche Applied Science, Indianapolis, IN). The tissue homogenates were clarified by centrifugation at 14,000 × *g* for 10 minutes. Protein concentrations of the resulting supernatants were determined using the bicinchoninic acid protein assay kit (Fisher Scientific, Houston, TX). The presence of human AβPP in the forebrain tissue homogenates was determined by immunoblotting using monoclonal antibody P2-1, which is specific for human AβPP.^33^ Bands corresponding to human AβPP were visualized using a LICOR imaging system (Lincoln, NE).

### ELISA Measurements of Aβ Peptides

The levels of total Aβ40 and Aβ42 were determined by performing specific ELISAs for each peptide, as described.^34^^,^^35^ Briefly, brain hemispheres that were flash frozen were and pulverized in liquid nitrogen. To determine total Aβ peptide levels, pulverized brain tissues were suspended in 5 mol/L guanidine-HCl and 50 mmol/L Tris, pH 8.0, and rotated at room temperature for 3 hours. Samples were centrifuged, and the supernatant was collected and a sandwich ELISA was performed. Antibody reagents for the Aβ ELISAs were generously provided by Lilly Research Laboratories (Indianapolis, IN). In the sandwich ELISAs, Aβ40 and Aβ42 peptides were captured using the carboxyl-terminal specific antibodies m2G3 and m21F12, respectively, and biotinylated m3D6, specific for the N-terminus of human Aβ, was used for detection followed by streptavidin–horseradish peroxidase (Amdex RPN4401V; Fisher Scientific, Pittsburgh, PA). Plates were developed using KPL SureBlue (SeraCare, Milford, MA) and read using a Spectramax M2 plate reader (Molecular Devices, Sunnyvale, CA). Each sample lysate was measured in triplicate and compared with linear standard curves generated with known concentrations of specific human Aβ peptides.

### Immunohistochemical Analysis

Antigen retrieval was performed by treating the tissue sections with proteinase K (0.2 mg/mL) for 10 minutes at 22°C. Primary antibodies were detected with biotinylated secondary antibodies and visualized with staining using horseradish peroxidase–conjugated diaminobenzidine or detected fluorescently using Alexa Fluor 594–conjugated donkey anti-rabbit or Alexa Fluor 488–conjugated goat anti-mouse secondary antibodies (1:1000). Staining for fibrillar amyloid was performed either using Amylo-Glo, as described by the manufacturer (Biosensis Inc., Thebarton, SA, Australia) or staining with thioflavin S or Congo red. The following antibodies were used for immunohistochemical analysis: rabbit polyclonal antibody to collagen type IV to visualize cerebral vessels (1:100; ThermoFisher, Rockford, IL); goat polyclonal antibody to glial fibrillary acidic protein (1:250; ab53554; Abcam, Cambridge, UK) or ionized calcium-binding adapter molecule 1 (1:250; NB100-1028; Novus, Littleton, CO) used to identify astrocytes and microglia, respectively; rabbit monoclonal antibody to transforming growth factor (TGF)-β1 (1:200; AB_215715; Abcam); rat monoclonal red fluor 710 antibody to Ly6 (1:200; AB 242065; Abcam); and mouse mAb CP-13 was raised against paired helical filaments from AD brain and recognizes tau phosphorylated at serine 202 (1:200; a gift from Dr. Peter Davies, Litwin-Zucker Center for Research on Alzheimer's Disease at the Feinstein Institute, Manhasset, NY).

Cortical CAA and amyloid plaque frequency were quantified on the same set of systematically sampled serial brain tissue sections representing every 10th section spanning the cortex of each analyzed rat brain. Tissue sections were immunolabeled for collagen type IV to identify blood vessels and staining with thioflavin S to identify fibrillar vascular amyloid deposits and parenchymal plaques. Serial 10× stitched images of the stained cortex were collected with a Keyence BZ-X700 Fluorescence Microscope (Itasca, IL). The Analyze Particles function in ImageJ version 1.52V (*https://imagej.net*) was used to calculate the percentage vessel area occupied by thioflavin S staining to determine cortical CAA load or the percentage parenchymal area occupied by thioflavin S–labeled plaques to determine cortical plaque load.

### Protein Digestion and Analysis by Trapped Ion Mobility Spectrometry Time-of-Flight (TIMS-TOF)

Total protein from each sample was reduced, alkylated, and purified by chloroform/methanol extraction before digestion with sequencing-grade modified porcine trypsin (Promega, Madison, WI). One microgram of tryptic peptides was loading on Evotip Pure disposable trap columns using a manufacturer suggested protocol. EvoSep One was used to elute and deliver peptides using the 30 sample per day gradient, which provides 44.0 minutes of separation time at a flow rate of 500 nL/minute. Peptides were eluted using a gradient from 98:2 to 65:35 buffer A:B ratio. Peptides were separated on a reverse phase 15 cm × 150 μm Endurance column (EvoSep, Copenhagen, Denmark) containing ReproSil-Pur C18, 1.9 μm beads at 40°C. Eluted peptides were ionized by CaptiveSpray (1650 V) held at 180°C, followed by mass spectrometric analysis on an TimsTOF Pro 2 mass spectrometer (Bruker, Billerica, MA). Ion mobility was set from 0.85 to 1.30 V·s/cm^2^ with a ramp time of 100 milliseconds and the mass spectrometry 1 range from 100 to 1700 m/z. With a scan mode set to data independent acquisition with parallel accumulation-serial fragmentation (DIA-PASEF) in the positive polarity, the DIA-PASEF windows range was from 475 to 1000 m/z for various mobilities and from 0.85 to 1.27 V·s/cm^2^ for mass spectrometry 2 with a cycle time estimate of 0.95 seconds. The collision energy settings were 20.00 eV at 0.60 V·s/cm^2^ and 59.00 eV at 1.60 V·s/cm^2^.

Buffer A = 0.1% formic acid and 0.5% acetonitrile.

Buffer B = 0.1% formic acid and 99.9% acetonitrile.

### Data Processing

Raw spectral data were analyzed with Spectronaut software version 19.1.240806.62635 (Biognosys, Schlieren, Switzerland), as previously described,^29^^,^^36^, ^37^, ^38^, ^39^, ^40^ using Spectronaut factory defaults, except used Biognosys' iRT kit and PTM localization were deselected, and normalization strategy set to local. Analyses were conducted as three separate parallel analyses comparing each of the transgenic models rTg-D HEM, rTg-D HOM, and TgSD-AD against the WT rat cohort. Protein identification and quantification were performed by the Spectronaut Pulsar algorithm, referencing a newly formed in-house spectral library that included searches of raw spectral files from rat regional and whole brain tissue samples, maintaining a false discovery rate of 0.01 at the protein, peptide, and protein spectrum match levels. Spectronaut output raw protein intensities were converted to molar concentrations (pmol/mg total protein) according to the total protein approach.^41^ As done previously, an imputed baseline concentration was used for protein concentrations of zero (filtered by Spectronaut for low intensity) in individual samples based on the lowest calculated total protein approach concentration in each analysis. These imputed values were 0.001, 0.007, and 0.0006 pmol/mg in the rTg-D HEM, rTg-D HOM, and TgSD-AD analyses, respectively. Average molar concentrations were compared to identify differentially expressed proteins between WT rats and transgenic rats. Statistical significance between groups was determined by *t*-test. A Benjamini-Hochberg adjustment was applied to the resultant *P* values; however, this correction proved to be too restrictive, as it removed all meaningful changes from the rTg-D HEM group. Therefore, uncorrected *P* values were considered, although both raw and corrected *P* values are reported in all differentially expressed protein (DEPs) tables. DEPs were, therefore, defined as proteins with a ≥50% increase or ≥33% decrease in protein expression compared with WT rats with a *P* value of ≤0.05.

### Data Availability

Raw spectral data for the proteomic analysis can be accessed in the PRoteomics IDEntifications Database (PRIDE; *https://www.ebi.ac.uk/pride/login*, last accessed March 1, 2025) repository under the project accession: PXD061304, and token: oopOP0kZ29oa.

### Statistical Analysis

Statistical analyses were performed using GraphPad Prism version 10.1.2 (Dotmatics, Boston, MA). For the proteomic analyses, statistical significance was calculated by two-tailed unpaired *t*-test, where two experimental conditions were present. Two-way analysis of variance tests with Sidak multiple comparison *post hoc* test was used when more than two experimental groups were compared. All data are represented as means ± SD.

## Results

### rTg-D HOM Rats Develop More Robust Cerebral Amyloid Pathology Compared with rTg-D HEM Rats

**Supplemental Figure S1:**
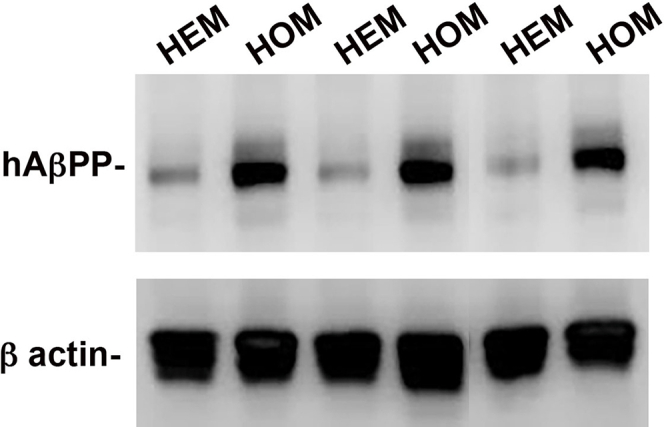
Immunoblot analysis of human amyloid β precursor protein (AβPP) (hAβPP) expression in total brain homogenates from rTg-D hemizygous (HEM) rats and rTg-D homozygous (HOM) rats. Immunoblot analysis of human AβPP expression in total brain homogenates from 12-month–old rats was performed using the anti-human AβPP mAbP2.1, as described in Materials and Methods. **Top panel:** Immunoblot for human AβPP. **Bottom panel:** Immunoblot for β-actin. rTg-HOM rats express higher levels of human AβPP compared with rTg-HEM rats.

Cohorts of rTg-D HEM and rTg-D HOM rats were aged 12 to 24 months, and their brains were harvested to measure the accumulation of cerebral amyloid pathology. As expected, rTg-D HOM rats exhibited higher levels of transgenic human AβPP in their brains compared with rTg-D HEM rats (Supplemental Figure S1). Consistent with this finding on AβPP levels, ELISA measurements showed that at all ages investigated rTg-D HOM rats accumulated much higher levels of Aβ40 and Aβ42 peptides in their brains compared with rTg-D HEM rats (Table 1). Two-way analysis of variance showed significant main effects for both genotype and age. For example, at 12 months, the rTg-D HOM rats accumulated approximately 45- and 28-fold higher levels of Aβ40 and Aβ42, respectively, compared with rTg-D HEM rats. As the animals aged further to 24 months, the rTg-D HEM rats accumulated appreciable amounts of Aβ40 and Aβ42, but the levels in the rTg-D HOM rats were still approximately 28- and 7-fold higher. At any age, the shorter Aβ40 species accounted for >90% of the total Aβ accumulating in the brains of either model.

**Table 1:** Cerebral Aβ40 and Aβ42 Levels in rTg-D HEM Rats and rTg-D HOM Rats

Genotype and age	Group size	Aβ40, mean ± SD, pg/mg brain	*P* value	Aβ42, mean ± SD, pg/mg brain	*P* value
HEM rTg-D 12M	*n* = 6	549 ± 997		70 ± 64	
HOM rTg-D 12M	*n* = 6	24,488 ± 14,320	NS	2003 ± 1338	NS
HEM rTg-D 18M	*n* = 6	11,744 ± 5978		1107 ± 393	
HOM rTg-D 18M	*n* = 5	179,422 ± 101,848	0.0001	19,654 ± 13,696	0.0001
HEM rTg-D 24M	*n* = 6	31,694 ± 20,983		2128 ± 1044	
HOM rTg-D 24M	*n* = 6	158,805 ± 94,860	0.0005	14,570 ± 8853	0.0019

Total Aβ40 and Aβ42 levels were determined in rat forebrains collected from cohorts of 12- to 24-month–old rTg-D HEM rats and rTg-D HOM rats using an enzyme-linked immunosorbent assay, as described in Materials and Methods. *P* values were assessed by two-way analysis of variance with Sidak multiple comparison test.

HEM, hemizygous; HOM, homozygous; M, months; NS, not significant.

**Figure 1:**
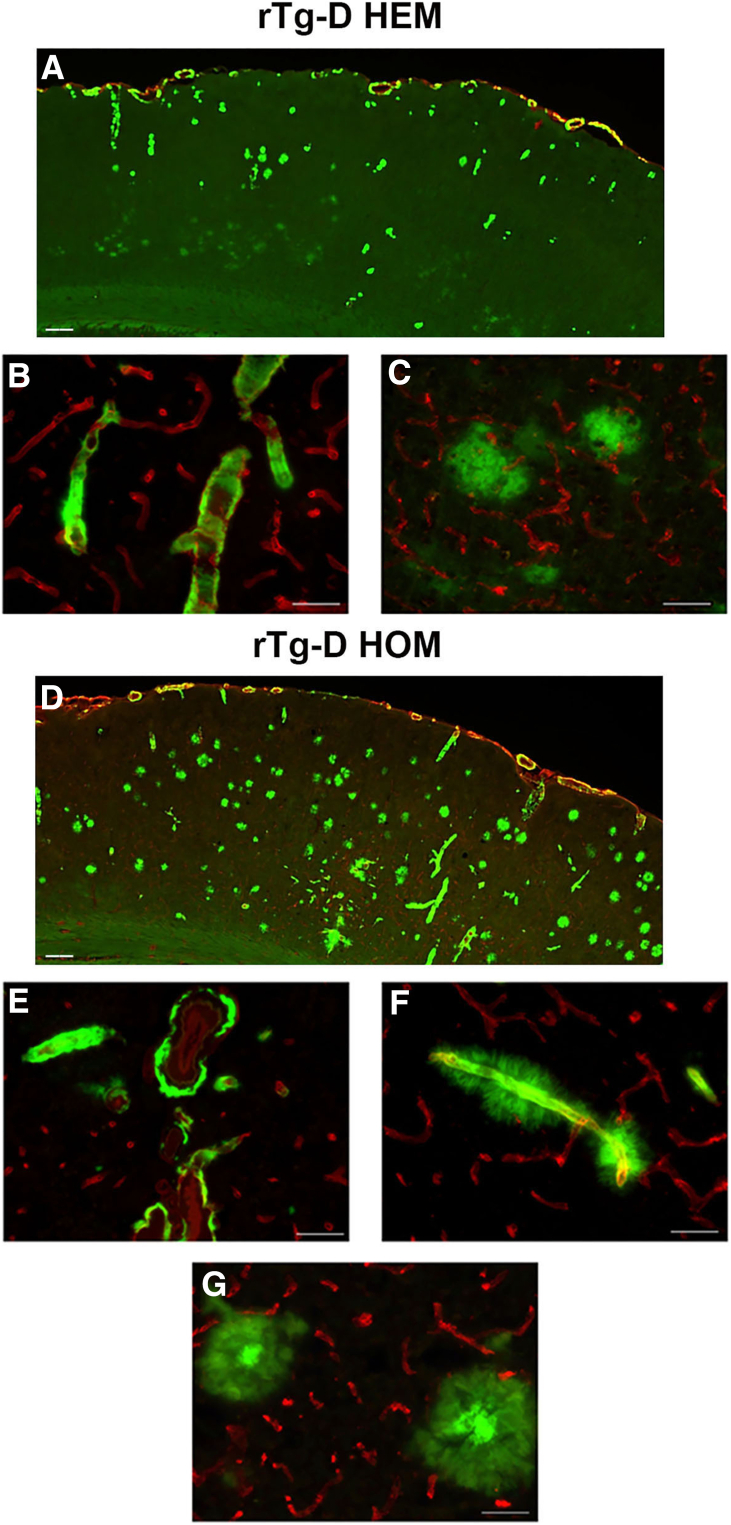
Cortical amyloid pathology in aged rTg-D hemizygous (HEM) and homozygous (HOM) rats. Representative coronal brain sections from 24-month–old rTg-D HEM rat (**A**) and 24-month–old rTg-D HOM rat (**D**) immunolabeled with an antibody to collagen IV to identify cerebral blood vessels (red) and stained with thioflavin S to visualize fibrillar amyloid (green) showing the presence of abundant of pial and cortical arteriolar cerebral amyloid angiopathy (CAA) in both models and numerous cortical plaques in the rTg-D HOM rats. Representative images from cortical arterioles with CAA (**B**, **E**, and **F**) and cortical parenchymal deposits (**C** and **G**). Scale bars: 1 mm (**A** and **D**); 50 μm (**B**, **C**, and **E**–**G**).

**Figure 2:**
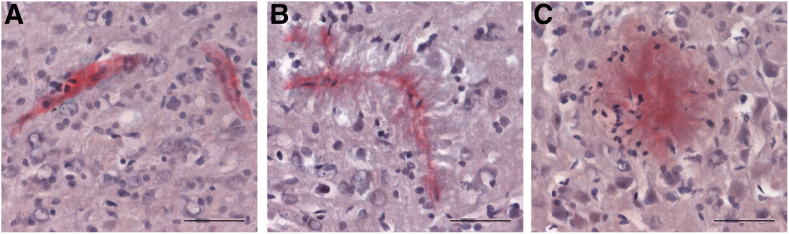
Cortical congophilic amyloid deposits in rTg-D homozygous (HOM) rats. Brain tissue from 24-month–old rTg-D HOM rats was stained with Congo red to identify fibrillar amyloid deposits. Cortical vessels presenting with fibrillar amyloid contained within the vessel wall (**A**); cortical vessel with dyshoric amyloid fibrils radiating out from the vessel (**B**); and cortical fibrillar amyloid plaque (**C**). Scale bar = 50 μm (**A**–**C**).

**Supplemental Figure S2:**
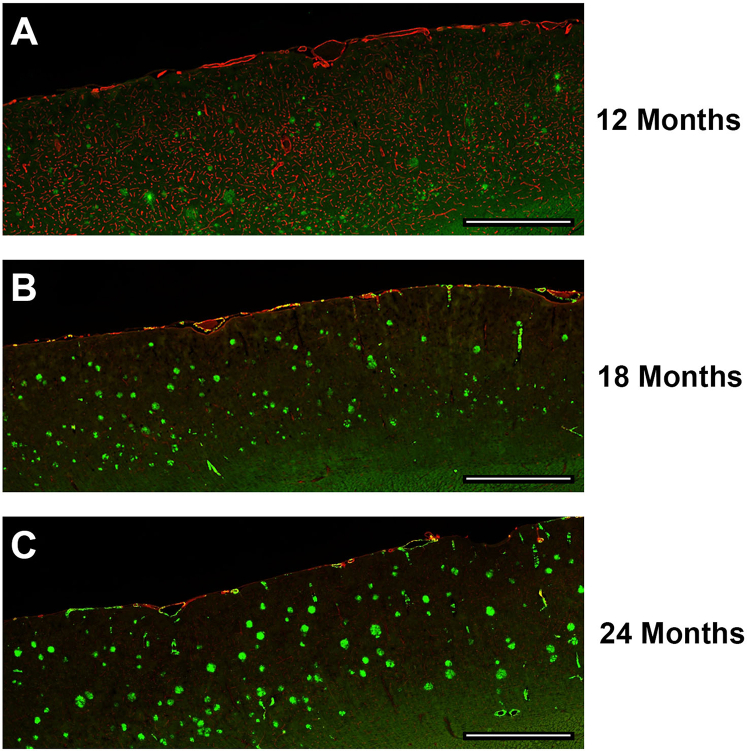
Progressive cortical amyloid pathology in rTg-D homozygous (HOM) rats. Representative coronal brain sections from increasingly aged rTg-D HOM rats immunolabeled with an antibody to collagen IV to identify cerebral blood vessels (red) and stained with thioflavin S to visualize fibrillar amyloid (green) showing the emergence and progression of cortical arteriolar cerebral amyloid angiopathy in numerous parenchymal plaques. rTg-D HOM rats aged 12 months (**A**), 18 months (**B**), and 24 months (**C**). Scale bar = 1 mm (**A**–**C**).

To evaluate the cerebral distribution of amyloid in rTgD HOM rats, brains were collected at 12, 18, and 24 months, and tissue sections were immunolabeled for collagen IV to visualize blood vessels and stained with thioflavin S to identify fibrillar Aβ deposits. Supplemental Figure S2 shows representative cortical regions of rTg-D HOM rats displaying the progression of cerebral amyloid pathology. Figure 1 shows a representative image of the cortex at 24 months from each line. rTg-D HEM rats had strong levels of CAA, particularly in the surface meningeal vessels and penetrating arterioles (Figure 1A). Examination of these deposits at higher magnification showed that rTg-D HEM rats presented with typical CAA type-2 with fibrillar amyloid contained within the vessel wall and some largely diffuse Aβ deposits in the parenchyma (Figure 1, B and C), as previously described.^28^^,^^29^ rTg-D HOM rats had even more robust levels of CAA, particularly in the intracortical vessels, and appearance of abundant parenchymal amyloid plaque pathology (Figure 1D). rTg-D HOM rats similarly presented with typical CAA type-2 (Figure 1E) but also had many vessels with dyshoric amyloid fibrils radiating out from the vessels (Figure 1F) and parenchymal amyloid plaques with a dense core (Figure 1G). Congo red staining of brain tissue sections from aged rTg-D HOM rats similarly showed examples of cortical arterioles with fibrillar amyloid restricted within the vessels (Figure 2A), dyshoric vascular amyloid (Figure 2B), and numerous congophilic parenchymal amyloid plaques (Figure 2C).

Percentage area fraction of vessels covered with vascular amyloid in the cortex, thalamus, and surface meningeal vessels of 18- and 24-month rTg-D HEM rats and rTg-D HOM rats was measured. The results presented in Table 2 show that at both ages and in each brain region, the CAA load was markedly higher in the rTg-D HOM rats compared with that in rTg-D HEM rats, consistent with the histologic images presented in Figure 1. The analysis of variance highlighted significant differences in all regions for both genotype and age. Similarly, the percentage area of cortical plaque coverage was >10-fold higher in the rTg-D HOM rats compared with that in the rTg-D HEM rats at either age (Table 3). Two-way analysis of variance showed significant effects for both genotype and age, whereas the percentage area of cortical plaque coverage increased much more robustly in rTg-D HOM rats when compared with rTg-D HEM rats.

**Table 2:** Regional CAA Load in rTg-D HEM Rats and rTg-D HOM Rats

Genotype, age, and brain region	Group size	% Vascular amyloid coverage	*P* value
HEM rTg-D 18M Cortex	*n* = 11	2.73 ± 2.67	
HOM rTg-D 18M Cortex	*n* = 6	13.53 ± 14.10	NS
HEM rTg-D 18M Thalamus	*n* = 11	3.30 ± 7.58	
HOM rTg-D 18M Thalamus	*n* = 6	14.90 ± 8.03	NS
HEM rTg-D 18M Meningeal	*n* = 11	6.50 ± 5.94	
HOM rTg-D 18M Meningeal	*n* = 6	36.72 ± 12.40	0.0001
HEM rTg-D 24M Cortex	*n* = 11	4.94 ± 6.90	
HOM rTg-D 24M Cortex	*n* = 7	27.51 ± 12.32	0.0001
HEM rTg-D 24M Thalamus	*n* = 11	6.35 ± 7.51	
HOM rTg-D 24M Thalamus	*n* = 7	30.77 ± 20.57	0.0006
HEM rTg-D 24M Meningeal	*n* = 11	16.82 ± 15.71	
HOM rTg-D 24M Meningeal	*n* = 7	52.20 ± 6.01	0.0001

The regional CAA load was determined in cohorts of 18- and 24-month–old rTg-D HEM rats and rTg-D HOM rats, as described in Materials and Methods. *P* values were assessed by two-way analysis of variance with Sidak multiple comparison test.

CAA, cerebral amyloid angiopathy; HEM, hemizygous; HOM, homozygous; M, months; NS, not significant.

**Table 3:** Cortical Parenchymal Plaque Load in rTg-D HEM Rats and rTg-D HOM Rats

Genotype and age	Group size	% Cortical plaque coverage	*P* value
HEM rTg-D 18M	*n* = 12	0.063 ± 0.037	
HOM rTg-D 18M	*n* = 6	1.112 ± 0.031	0.0001
HEM rTg-D 24M	*n* = 10	0.349 ± 0.238	
HOM rTg-D 24M	*n* = 7	2.901 ± 0.712	0.0001

The cortical parenchymal plaque load was determined in cohorts of 18- and 24-month–old rTg-D HEM rats and rTg-D HOM rats, as described in Materials and Methods. *P* values were assessed by two-way analysis of variance with Sidak multiple comparison test.

HEM, hemizygous; HOM, homozygous; M, months.

**Supplemental Figure S3:**
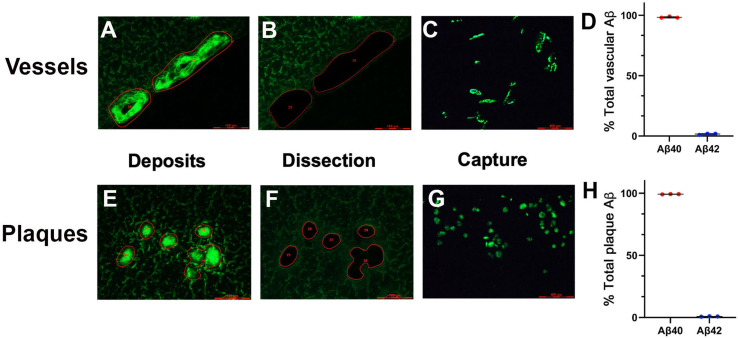
Amyloid β (Aβ) enzyme-linked immunosorbent assay (ELISA) analysis of isolated vascular amyloid and parenchymal plaque deposits in rTg-D homozygous (HOM) rats. Laser capture microdissection (LCM) was used to collect vascular amyloid deposits (**A**–**C**) and parenchymal amyloid plaque deposits (**E**–**G**) from brain tissue sections from 24-month–old rTg-D HOM rats. Three hundred vascular and plaque amyloid deposits collected from each rat were subjected to ELISA analysis for Aβ40 and Aβ42, as described in Materials and Methods. The percentage total of Aβ40 (red) and Aβ42 (blue) from collected vascular amyloid (**D**) and parenchymal plaque amyloid (**H**). Both vascular and parenchymal amyloid deposits in rTg-D HOM rats contain approximately 95% Aβ40 and approximately 5% Aβ42. Red outlining indicates areas with amyloid deposits collected by LCM. Red numbering indicates the specific amyloid deposit collected. *n* = 3 rats (**A**–**C** and **E**–**G**). Scale bars: 100 μm (**A**, **B**, **E**, and **F**); 400 μm (**C** and **G**).

To determine the Aβ peptide composition of the cerebral vascular and parenchymal plaque amyloid deposits in rTg-D HOM rats, laser capture microdissection was performed for each type of deposit and ELISA analysis was conducted for Aβ40 and Aβ42. Supplemental Figure S3 shows that both the cerebral vascular and parenchymal plaque amyloid deposits in the rTg-D HOM rats are composed of approximately 95% shorter Aβ40 peptide, similar to previous findings in rTg-D HEM rats.^28^ Taken together, these findings show that rTg-D HOM rats accumulate much higher levels of amyloid, primarily composed of Aβ40, in the form of CAA and parenchymal amyloid plaques compared with rTg-D HEM rats.

### rTg-D HOM Rats Exhibit Increased Activated Astrocytes and Microglia and Phosphorylated Tau in Response to Dyshoric Vascular and Parenchymal Plaque Amyloid

**Figure 3:**
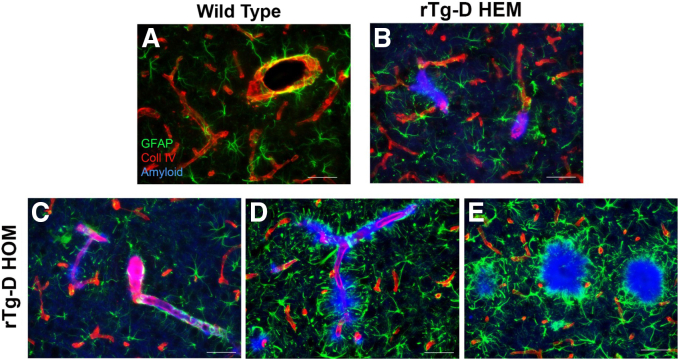
Astrocytic responses in rTg-D hemizygous (HEM) and homozygous (HOM) rats. Brain tissue from 24-month–old wild-type (WT) rats (**A**), rTg-D HEM rats (**B**), or rTg-D HOM rats (**C**–**E**) was immunolabeled with antibodies to glial fibrillary acidic protein (GFAP) to identify astrocytes (green) or collagen (Coll) IV to identify cerebral blood vessels (red) and stained with Amylo-Glo to visualize fibrillar amyloid (blue). Normal perivascular distribution of astrocytes is observed around unaffected cortical cerebral vessels in WT rats (**A**) or with typical cerebral amyloid angiopathy (CAA) in rTg-D HEM rats (**B**) or with typical CAA in rTg-D HOM rats (**C**) with fibrillar amyloid restricted within the vessel wall. In contrast, rTg-D HOM rats presenting with cortical dyshoric vascular amyloid (**D**) or cortical fibrillar parenchymal plaques (**E**) exhibited a marked increase and activation of astrocytes engaged with fibrillar amyloid. Scale bar = 50 μm (**A**–**E**).

Typical CAA type-2 restricted to the cerebral vessel wall in rTg-D HEM rats does not elicit robust perivascular astrocytic or microglial responses.^28^^,^^29^ This was confirmed here, when CAA type-2 restricted within the cerebral vessel wall of rTg-D HEM rats (Figure 3B) exhibited a normal distribution of perivascular astrocytes like aged WT rats (Figure 3A). Similarly, when CAA type-2 was confinded to the cerebral vessel wall of rTg-D HOM rats, astrocytes displayed a normal distribution (Figure 3C). In contrast, when dyshoric vascular amyloid extended into the surrounding parenchyma or accumulated as parenchymal amyloid plaques, a robust astrocytic response was observed (Figure 3, D and E).

**Figure 4:**
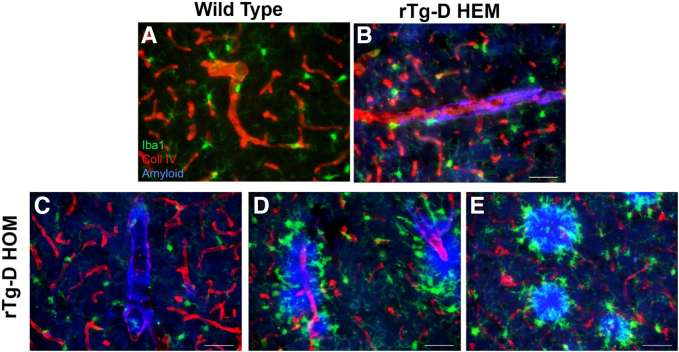
Microglial responses in rTg-D hemizygous (HEM) and homozygous (HOM) rats. Brain tissue from 24-month–old wild-type (WT) rats (**A**), rTg-D HEM rats (**B**), or rTg-D HOM rats (**C**–**E**) was immunolabeled with antibodies to ionized calcium-binding adapter molecule 1 (Iba1) to identify microglia (green) or collagen (Coll) IV to identify cerebral blood vessels (red) and stained with Amylo-Glo to visualize fibrillar amyloid (blue). Normal distribution of microglia in their resting ramified state is observed around unaffected cortical vessels in WT rats (**A**) or with typical cerebral amyloid angiopathy (CAA) in rTg-D HEM rats (**B**) or with typical CAA in rTg-D HOM rats (**C**) with fibrillar amyloid restricted within the vessel wall. In contrast, rTg-D HOM rats presenting with cortical dyshoric vascular amyloid (**D**) or cortical fibrillar parenchymal plaques (**E**) exhibited a marked increase and activation of microglia engaged with fibrillar amyloid. Scale bar = 50 μm (**A**–**E**).

Similarly, when CAA type-2 was confined to the cerebral vessel walls in rTg-D HEM rats, microglia exhibited a normal distribution and remained in a resting surveillance mode with extended processes (Figure 4B), resembling the aged WT rats (Figure 4A). Similarly, when CAA type-2 was confined to the cerebral vessel wall of rTg-D HOM rats, there was a normal distribution of resting state microglia (Figure 4C). However, when dyshoric vascular amyloid extended into the adjacent parenchyma or accumulated as parenchymal amyloid plaques, there was a marked increase in activated microglia engaged with the parenchymal amyloid (Figure 4, D and E).

**Figure 5:**
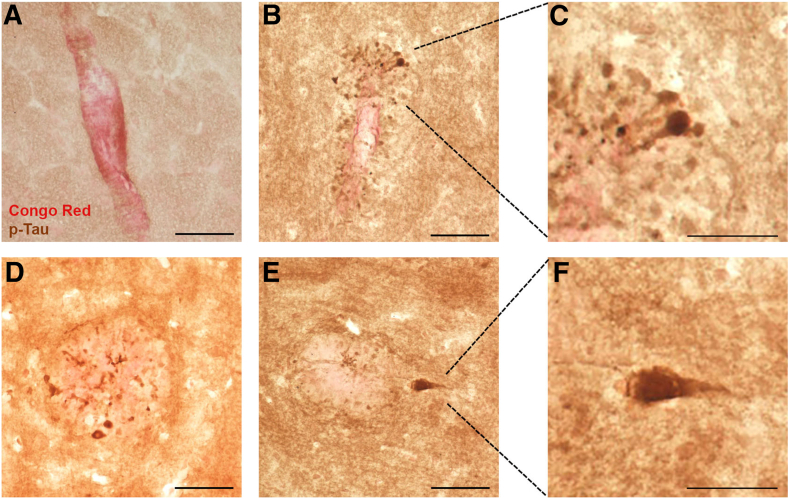
Phosphorylated tau (p-Tau) pathology in rTg-D homozygous (HOM) rats. Brain tissue from 24-month–old rTg-D HOM rats was immunolabeled with CP-13 monoclonal antibody to visualize phosphorylated tau (brown) and stained with Congo red to identify fibrillar amyloid deposits (red) in the cortex. **A:** rTg-D HOM rats with typical cerebral amyloid angiopathy with fibrillar amyloid restricted within the vessel wall showed no phosphorylated tau labeling. **B**, **D**, and **E:** In contrast, rTg-D HOM rats presenting with congophilic dyshoric vascular amyloid (**B**) or fibrillar parenchymal plaques (**D** and **E**) in the cortex exhibited robust phosphorylated tau labeling in dystrophic neurites associated with fibrillar amyloid. **B** and **E:** The **dotted lines** indicate the enlarged areas displayed in **C** and **F**, respectively. **C** and **F:** Occasional phosphorylated tau-positive, tangle-like structures were associated with dyshoric vascular amyloid (**C**) and cortical amyloid plaques (**F**). Scale bars: 50 μm (**A**, **B**, **D**, and **E**); 25 μm (**C** and **F**).

Rats are a valuable model to investigate ADRD pathology because they express the full complement of tau isoforms and can develop phosphorylated tau pathology in response to parenchymal amyloid deposition.^31^ Given that rTg-D HOM rats exhibit parenchymal amyloid pathology in the form of congophilic dyshoric vascular amyloid and parenchymal amyloid plaques, whether they develop associated phosphorylated tau pathology was assessed next. In rTg-D HOM rats, CAA type-2 restricted to the cerebral vessel wall showed no signs of perivascular phosphorylated tau pathology (Figure 5A). However, when dyshoric vascular amyloid extended into the adjacent parenchyma, phosphorylated tau accumulated in surrounding dystrophic neurites (Figure 5B), with occasional formation of tangle-like structures (Figure 5C). Phosphorylated tau labeling was present in 12.2% ± 2.0% of the area occupied by dyshoric vascular amyloid. Similarly, congophilic amyloid plaques in the brain parenchyma of rTg-D HOM rats also exhibited phosphorylated tau accumulating in surrounding dystrophic neurites (Figure 5, D and E) along with the emergence of tangle-like structures (Figure 5F). In these plaques, phosphorylated tau labeling was present in 3.8% ± 0.2% of the area occupied by plaque amyloid, significantly less than in dyshoric vascular amyloid (*P* < 0.002). Together, these findings indicate that parenchymal amyloid deposition in rTg-D HOM rats, in the forms of dyshoric vascular amyloid and parenchymal plaques, elicits strong astrocytic and microglial activation and adjacent phosphorylated tau pathology, which is more pronounced in areas surrounding dyshoric vascular amyloid.

### Differential Proteomic Analysis in rTg-D HEM and HOM Rats

**Figure 6:**
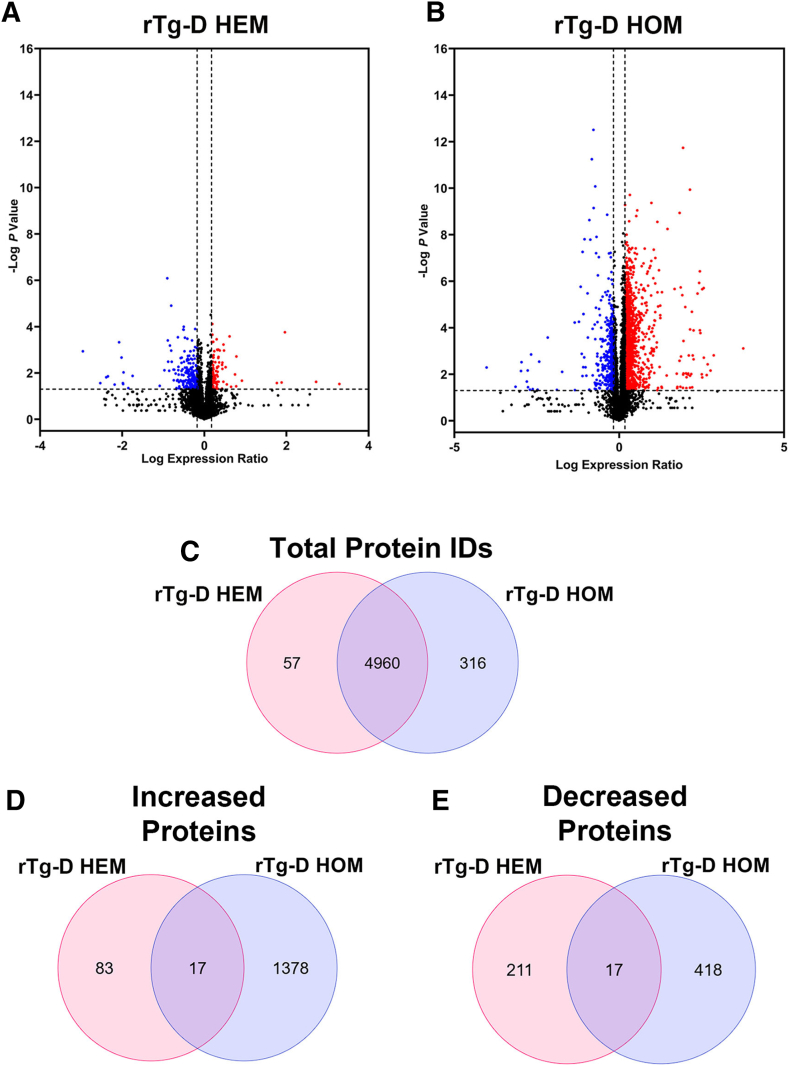
Differentially expressed proteins (DEPs) in aged rTg-D hemizygous (HEM) and homozygous (HOM) rat brains. **A** and **B:** Volcano plots generated from log fold change and –log *P* values for all proteins analyzed in rTg-D HEM (**A**) and rTg-D HOM (**B**) rat brains. Points representing proteins ≥50% increase (indicated by **right vertical dotted line**) and proteins ≥33% decrease (indicated by **left vertical dotted line**), *P* ≤ 0.05 (indicated by **horizontal dotted line**) relative to wild-type rats are shown in red and blue, respectively. **C:** Venn diagram showing the numbers of unique and common proteins detected in rTg-D HEM and rTg-D HOM rats by analyses in Spectronaut. **D:** Venn diagram showing the numbers of unique and common significantly increased DEPs detected in rTg-D HEM and rTg-D HOM rats compared with wild-type rats. **E:** Venn diagram showing the numbers of unique and common significantly decreased DEPs detected in rTg-D HEM and rTg-D HOM rats compared with wild-type rats. ID, identifier.

To further investigate molecular changes contributing to the common and distinct pathologic profiles of rTg-D HEM and rTg-D HOM rats, comparative discovery proteomic analysis was conducted on whole brain tissue from aged cohorts of each line. Differential proteomic analysis of 18- to 24-month rTg-D HEM and HOM rat whole brain tissue was conducted in parallel analyses compared with whole brain tissue obtained from a WT rat cohort aged to 12 months, as described in Materials and Methods. DEPs were defined as proteins with a ≥50% increase or ≥33% decrease compared with WT protein expression, with *P* ≤ 0.05. Volcano plots of all identified proteins for the rTg-D HEM rat and rTg-D HOM rat analyses are displayed in Figure 6, A and B, with 5017 and 5276 proteins analyzed and quantified in the rTg-D HEM and rTg-D HOM rats, respectively, with a total of 4960 identified proteins shared between both analyses (Figure 6C). A total of 83 and 1378 proteins were uniquely increased in the rTg-D HEM rats and rTg-D HOM rats, respectively, with 17 proteins shared between the two lines (Figure 6D), including amyloid precursor protein (APP), apolipoprotein E (APOE), claudin 10 (CLDN10), cathepsin Z (CTSZ), and high temperature requirement A serine protease 1 (HTRA1). On the other hand, 211 and 418 proteins were uniquely decreased in the rTg-D HEM rats and rTg-D HOM rats, respectively, with 17 proteins shared between the two lines (Figure 6E). All DEPs from the rTg-D HEM rat and rTg-D HOM rat lines are listed in Supplemental Tables S1 and S2, respectively, and lists of the shared increased and decreased DEPs are provided in Supplemental Table S3. It is not surprising that the rTg-D HOM rats displayed significantly more DEPs than the rTg-D HEM rats as the pathology, including glial cell responses, neuroinflammation, as well dyshoric amyloid and fibrillar plaque pathology, is far more robust in the former. Furthermore, the rTg-D HEM rats exhibit greater variability in amyloid load compared with the rTg-D HOM rats, resulting in fewer statistically significant differences compared to WT controls. Although 17 proteins were shared between the rTg-D HEM and rTg-D HOM groups, this overlap represents a minority of the DEPs identified in either line. This limited overlap perhaps indicates that the observed proteomic changes in the rTg-D HOM rats are dominated by the dyshoric and parenchymal plaque pathology accompanied by the robust associated glial responses.

### Ingenuity Pathway Analysis of rTg-D HEM and HOM DEPs

**Figure 7:**
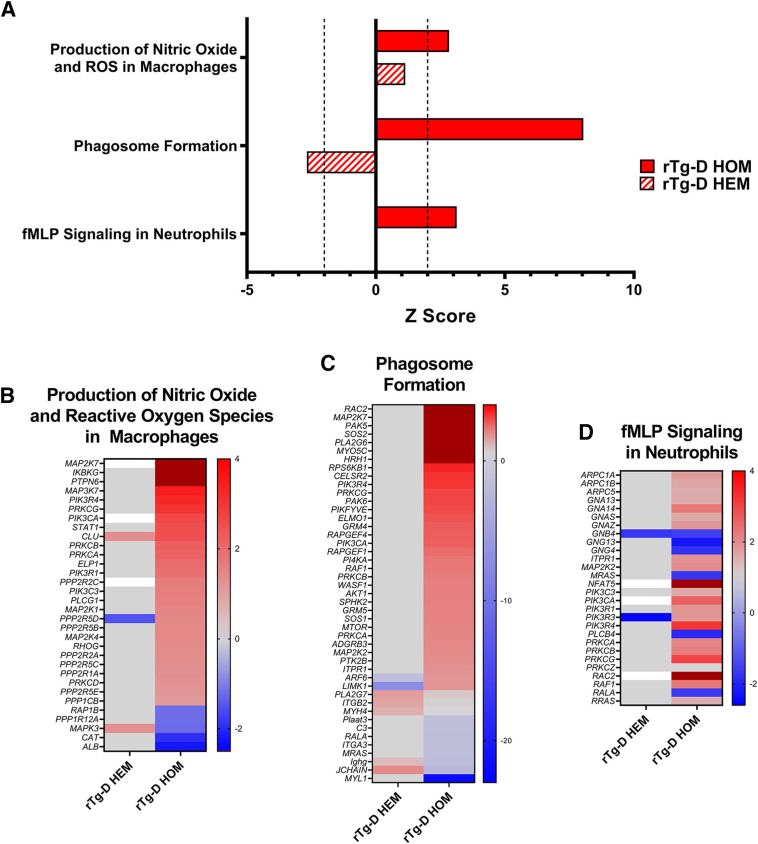
Summary of Ingenuity Pathway Analyses for rTg-D hemizygous (HEM) and rTg-D homozygous (HOM) rats. **A:** Select pathways predicted to be activated (z score >2) or inhibited (z score <–2) in rTg-D HEM and rTg-D HOM rat brains, with **dashed lines** at z = 2 and –2 indicating significantly altered pathways. **B**–**D:** Heat maps depicting differential expression relative to wild-type expression of proteins in the rTg-D HEM rat brains from the production of nitric oxide and reactive oxygen species (ROS) in macrophages (**B**), phagosome formation (**C**), and *N-*formyl-methionyl-leucyl-phenylalanine (fMLP) signaling in neutrophils (**D**) pathways with corresponding rTg-D HOM expression. Red indicates increased expression, blue decreased expression, gray no significant change, and white not detected, with color intensity relative to fold change.

To provide mechanistic context to the numerous observed DEPs, comparative pathway enrichment and activation analysis was performed using Ingenuity Pathway Analysis (IPA; Qiagen, Hilden, Germany) of the cerebral proteomics results from the rTg-D HEM and rTg-D HOM cohorts. Beyond enrichment analysis, IPA uses directional expression changes of proteins upstream and downstream of protein regulators, and in canonical pathway cascades to predict activation or inhibition of regulators and pathways with significance given in z scores.^42^ Figure 7A depicts the z scores of select activated pathways from the comparative analysis in either the rTg-D HEM or rTg-D HOM cohorts. Several pathways were predicted as significantly activated in the rTg-D HOM rats but not the rTg-D HEM rats, including production of nitric oxide and reactive oxygen species (ROS) in macrophages (z = 2.837), phagosome formation (z = 8.041), and *N-*formyl-methionyl-leucyl-phenylalanine (fMLP) signaling in neutrophils (z = 3.130) (Figure 7A). Heat maps of DEPs from these pathways in the rTg-D HOM rats, with their corresponding expression in the rTg-D HEM rats, are depicted in Figure 7, B–D. Phagosome formation and ROS and nitric oxide production contribute to inflammatory responses of immune cells, such as neutrophils or macrophages,^43^^,^^44^ but also microglia,^45^ and despite not producing large amounts of ROS, astrocytes become phagocytic following brain ischemia and for debris clearance.^46^ As neither of these pathways are activated in the rTg-D HEM model, this is likely indicative of the robust immune response elicited by the dyshoric amyloid and parenchymal plaques present in the rTg-D HOM rats, and perhaps further suggests involvement of immune cells in conjunction with brain resident glial cells. fMLP is a neutrophil chemoattractant promoting swarming of neutrophils^47^ derived from mitochondrial proteins and can be released following cell death.^48^ The fMLP signaling in neutrophils could indicate neutrophil swarming in response to the presence of dyshoric amyloid and fibrillar plaque pathology (see below).

### rTg-D HOM Rats Share Parenchymal Amyloid-Associated Changes with TgSD-AD Rats

**Figure 8:**
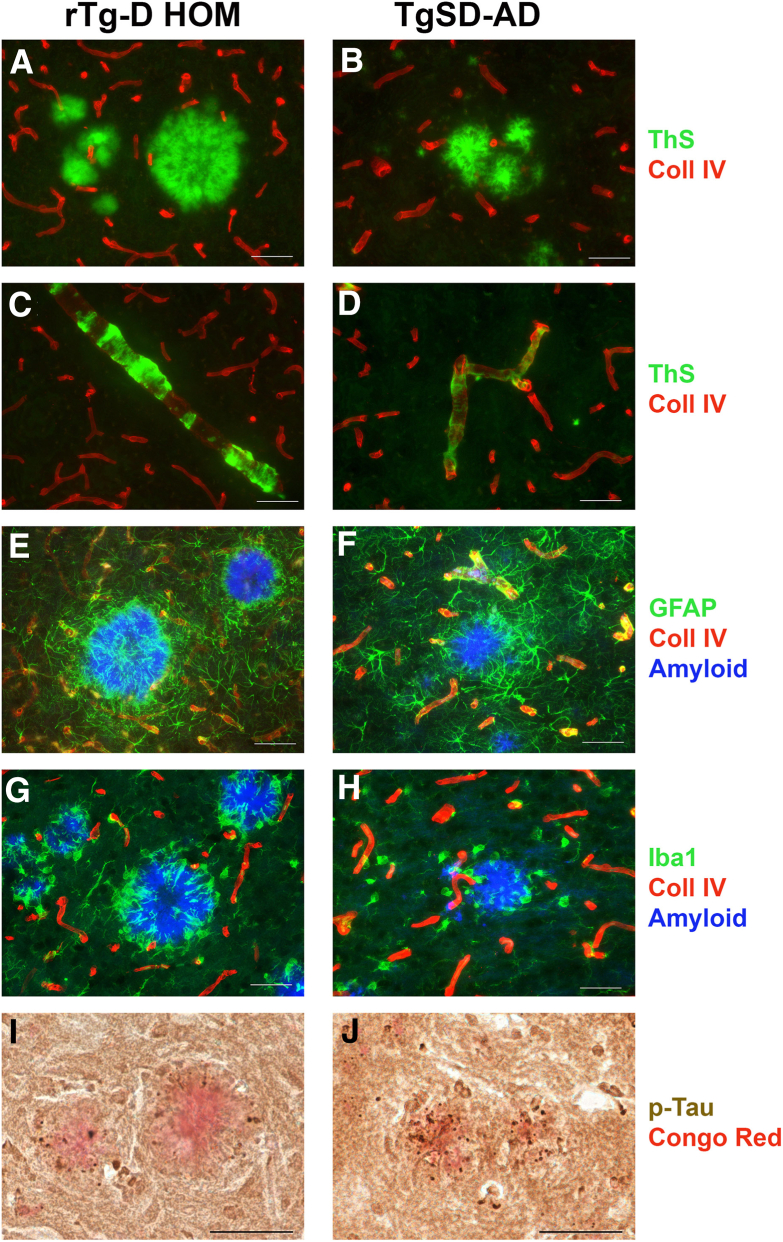
Comparison of cortical amyloid pathologies in rTg-D homozygous (HOM) rats and transgenic Sprague-Dawley–Alzheimer disease (TgSD-AD) rats. **A**–**D:** Brain tissues from 24-month–old rTg-D HOM rats and TgSD-AD rats were immunolabeled with antibodies to collagen (Coll) IV to identify cerebral blood vessels (red) and stained with thioflavin S (ThS) to identify fibrillar amyloid (green) showing cortical parenchymal amyloid plaques (**A** and **B**) and cortical arteriolar amyloid deposits (**C** and **D**). **E**–**H:** Immunolabeling with antibodies to glial fibrillary acidic protein (GFAP) to identify astrocytes (green; **E** and **F**) or to ionized calcium-binding adapter molecule 1 (Iba1) to identify microglia (green; **G** and **H**) and collagen IV to identify cerebral blood vessels (red) and stained with Amylo-Glo to visualize fibrillar cortical parenchymal amyloid plaques (blue). **I** and **J:** Immunolabeling with CP-13 monoclonal antibody to visualize phosphorylated tau (p-Tau; brown) and stained with Congo red to identify fibrillar cortical parenchymal amyloid plaques (red). Scale bar = 50 μm (**A**–**J**).

**Supplemental Figure S4:**
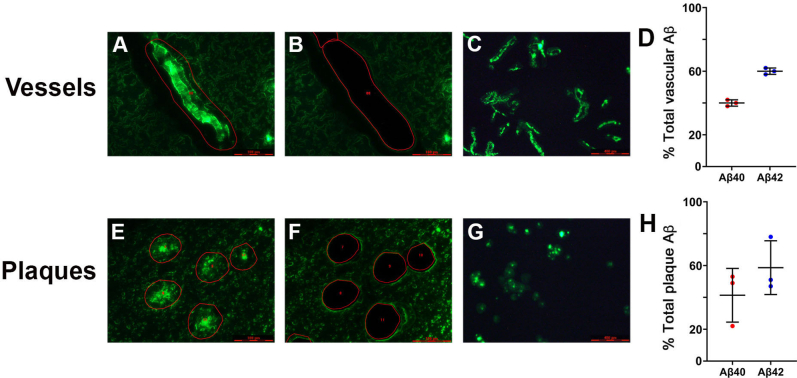
Amyloid β (Aβ) enzyme-linked immunosorbent assay (ELISA) analysis of isolated vascular amyloid and parenchymal plaque deposits in transgenic Sprague-Dawley–Alzheimer disease (TgSD-AD) model rats. Laser capture microdissection (LCM) was used to collect vascular amyloid deposits (**A**–**C**) and parenchymal amyloid plaque deposits (**E**–**G**) from brain tissue sections from 24-month–old TgSD-AD rats. Three hundred vascular and plaque amyloid deposits collected from each rat were subjected to ELISA analysis for Aβ40 and Aβ42, as described in Materials and Methods. The percentage total of Aβ40 (red) and Aβ42 (blue) from collected vascular amyloid (**D**) and parenchymal plaque amyloid (**H**). Both vascular and parenchymal amyloid deposits in TgSD-AD rats contain approximately 40% Aβ40 and approximately 60% Aβ42. Red outlining indicates areas with amyloid deposits collected by LCM. Red numbering indicates the specific amyloid deposit collected. *n* = 3 rats (**A**–**C** and **E**–**G**). Scale bars: 100 μm (**A**, **B**, **E**, and **F**); 400 μm (**C** and **G**).

The abundance of parenchymal amyloid plaque-associated pathologies in rTg-D HOM rats is reminiscent of AD pathologies. Therefore, comparisons were performed of the cerebral pathologies of rTg-D HOM rats with a rat model of AD-like pathologies. For this comparison, the TgF344-AD that was backcrossed onto a SD background for 10 generations and designated TgSD-AD was used.^32^ Because both the rTg-D HEM and HOM rat lines are also on a SD background, this facilitates meaningful comparisons between the different rat strains. Figure 8, A and B, shows that both rTg-D HOM rats and TgSD-AD rats develop cortical parenchymal fibrillar amyloid plaques, although they tended to be larger in rTg-D HOM rats. Similarly, both rat lines present with CAA type-2 (Figure 8, C and D), although the vascular amyloid deposition was more extensive in the rTg-D HOM rats. In contrast to rTg-D HOM rats, TgSD-AD rats did not present with any detectable dyshoric vascular amyloid. To determine the Aβ peptide composition of the cerebral vascular and parenchymal plaque amyloid deposits in TgSD-AD rats, laser capture microdissection was performed for each type of deposit and ELISA analysis was conducted for Aβ40 and Aβ42. Supplemental Figure S4 shows that both the cerebral vascular and parenchymal plaque amyloid deposits in the TgSD-AD rats contain much higher amounts of Aβ42 (approximately 60%) compared with similar deposits in rTg-D HOM rats (approximately 5%) shown in Supplemental Figure S3. Cortical parenchymal amyloid plaques were surrounded by activated astrocytes (Figure 8, E and F) and activated microglia (Figure 8, G and H) in both models. Lastly, congophilic parenchymal amyloid plaques in both models were decorated with labeling for phosphorylated tau in surrounding dystrophic neurites (Figure 8, I and J) at comparable levels (3.8% ± 0.2% for rTg-D HOM plaques versus 4.2% ± 0.3% for TgSD-AD plaques). Together, these findings show that rTg-D HOM rats and TgSD-AD rats develop similar cerebral amyloid pathologies with associated glial and phosphorylated tau responses, although the cerebral amyloid deposits in the latter contain much higher levels of Aβ42.

### Differential Proteomic Analysis in TgSD-AD Rats

**Figure 9:**
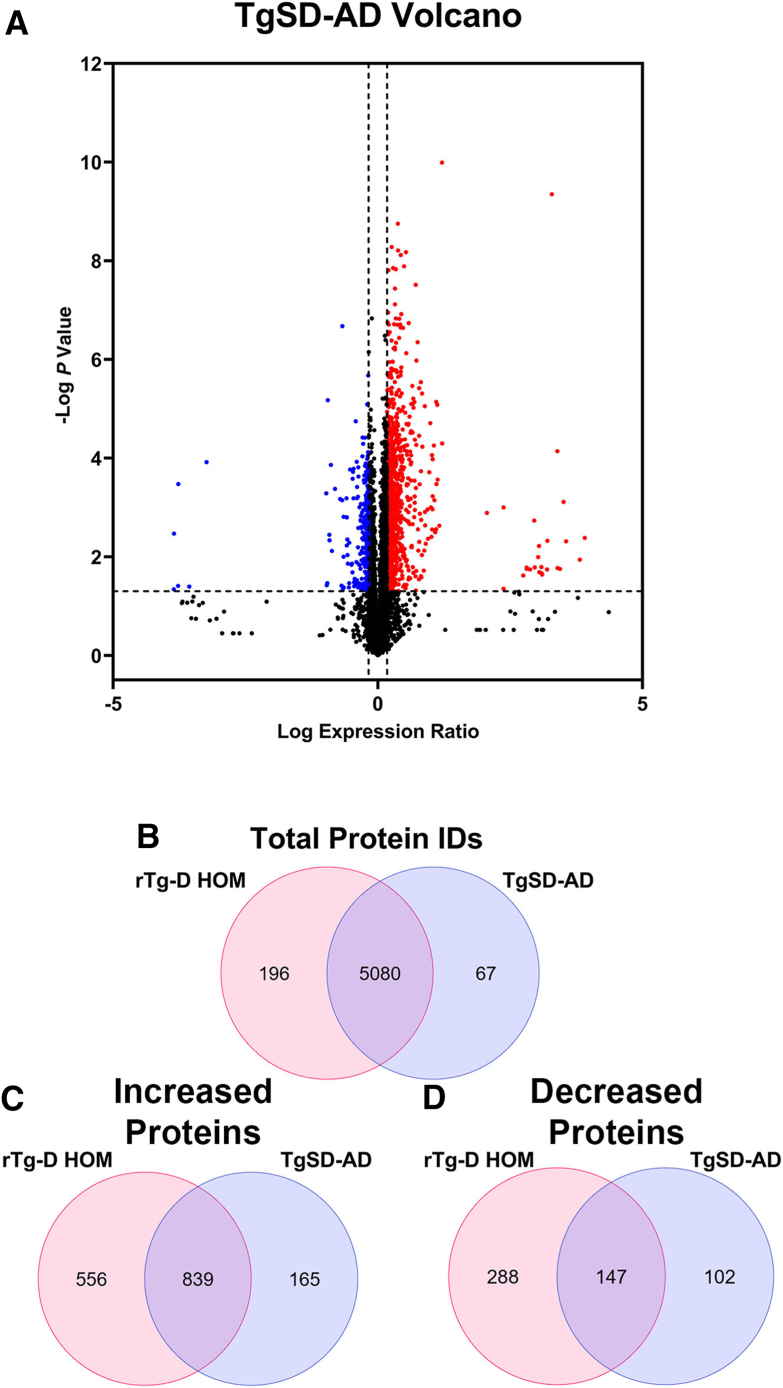
Differentially expressed proteins (DEPs) in aged transgenic Sprague-Dawley–Alzheimer disease (TgSD-AD) rat brains. **A:** Volcano plot generated from log fold change and –log *P* values for all proteins analyzed in TgSD-AD rat brains. Points representing proteins ≥50% increase (indicated by **right vertical dotted line**) and proteins ≥33% decrease (indicated by **left vertical dotted line**), *P* ≤ 0.05 (indicated by **horizontal dotted line**) relative to wild-type (WT) rats are shown in red and blue, respectively. **B:** Venn diagram showing the numbers of unique and common proteins detected in rTg-D homozygous (HOM) rats and TgSD-AD analyses in Spectronaut. **C:** Venn diagram showing the numbers of unique and common significantly increased DEPs detected in rTg-D HOM rats and TgSD-AD rats compared with WT rats. **D:** Venn diagram showing the numbers of unique and common significantly decreased DEPs detected in rTg-D HOM rats and TgSD-AD compared with WT rats. ID, identifier.

Because of their similar pathologies, differential proteomic analysis of the TgSD-AD rats was next conducted for comparison with the rTg-D HOM rats. As with the rTg-D HEM rats, the proteomic analysis of rTg-D HOM rats and TgSD-AD rats used whole brain tissue and was conducted in parallel analyses compared with whole brain tissue from the same WT rat cohort, and differentially expressed proteins were determined by the same criteria. A volcano plot depicting all the analyzed proteins in the TgSD-AD model is depicted in Figure 9A. All DEPs in the TgSD-AD line are listed in Supplemental Table S4. The TgSD-AD rats and rTg-D HOM rats were compared, and analyses displayed great similarity in total protein identifications (Figure 9B). Interestingly, although some differences existed, the vast majority (839) of the significantly elevated proteins were shared between the two models (Figure 9C), with 147 reduced proteins shared between the TgSD-AD rats and rTg-D HOM rats as well (Figure 9D). Lists of the shared increased and decreased DEPs in rTg-D HOM rats and TgSD-AD rats are provided in Supplemental Table S5. Thus, despite arising from distinct Aβ species with the rTg-D HOM rats depositing E22Q Dutch mutant Aβ40 and TgSD-AD rats accumulating WT Aβ42, the plaque pathology in both models appears to promote similar proteomic changes in the brain.

### Ingenuity Pathway Analysis of DEPs in rTg-D HOM and TgSD-AD Rat Brains

**Figure 10:**
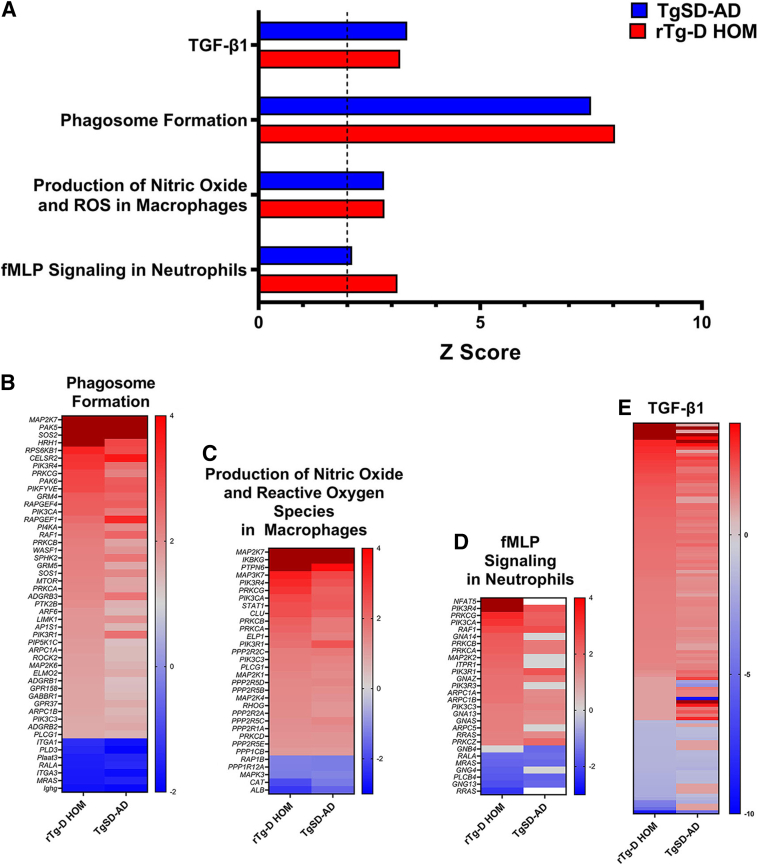
Summary of Ingenuity Pathway Analyses for rTg-D homozygous (HOM) and transgenic Sprague-Dawley–Alzheimer disease (TgSD-AD) rats. **A:** Select pathways predicted to be activated (z score >2) in rTg-D HOM and TgSD-AD rat brains, with **dotted line** at z = 2 to indicate significantly activated pathways. **B**–**E:** Heat maps depicting differential expression relative to wild-type expression of proteins in the rTg-D HOM rat brains from the phagosome formation (**B**), production of nitric oxide and reactive oxygen species (ROS) in macrophages (**C**), *N-*formyl-methionyl-leucyl-phenylalanine (fMLP) signaling in neutrophils (**D**), and transforming growth factor (TGF)-β1 network (**E**) with corresponding TgSD-AD expression. Red indicates increased expression, blue decreased expression, gray no significant change, and white not detected, with color intensity relative to fold change. The list of all differentially expressed proteins in the TGF-β1 pathway are provided in Supplemental Table S6.

**Figure 11:**
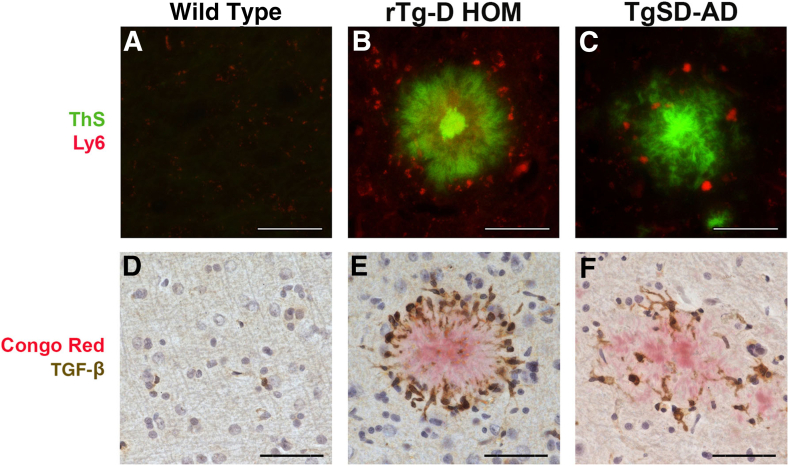
Cortical parenchymal plaque-associated Ly6 and transforming growth factor (TGF)-β1 labeling in rTg-D homozygous (HOM) rats and transgenic Sprague-Dawley–Alzheimer disease (TgSD-AD) rats. Brain tissue from 24-month–old wild-type rats, rTg-D HOM rats, and TgSD-AD rats was stained with thioflavin S (ThS) to identify cortical fibrillar amyloid plaques (green), and neutrophils were immunolabeled with an antibody to Ly6 (red; **A**–**C**) or stained with Congo red to identify fibrillar amyloid plaques (red) and immunolabeled with an antibody to TGF-β1 (brown; **D**–**F**). Scale bar = 50 μm (**A**–**F**).

Comparative IPA analysis of the DEPs in the TgSD-AD rats and rTg-D HOM rats was conducted to identify mechanistically relevant proteins from the large number of common DEPs. Not surprisingly, the two models shared significant similarity in predicted activated pathways, including those highlighted for rTg-D HOM rats above in Figure 7, such as phagosome formation (z = 7.500), production of nitric oxide and reactive oxygen species in macrophages (z = 2.828), fMLP signaling in neutrophils (z = 2.111), as well as the TGF-β1 network (z = 3.193 and 3.351 for rTg-D HOM and TgSD-SD, respectively) (Figure 10A). Heat maps of DEPs from these pathways in the rTg-D HOM rats, with their corresponding expression in the TgSD-AD rats, are depicted in Figure 10, B–E. As mentioned above, phagosome formation and ROS and nitric oxide production contribute to inflammatory responses of immune cells, such as neutrophils or macrophages, microglia, and astrocytes. Not only are they commonly predicted as activated by IPA, but also to similar degrees, despite not being activated in the rTg-D HEM rats, further suggesting that this is associated with the inflammatory response to the presence of parenchymal plaque pathology robustly presented in both the rTg-D HOM and TgSD-AD rats. The predicted activation of fMLP signaling in both the rTg-D HOM and TgSD-AD rats suggests neutrophil swarming occurs in the brain parenchyma in response to plaque amyloid pathology. Indeed, Figure 11, B and C, shows the presence of Ly6g-positive neutrophils around the periphery of cortical fibrillar amyloid plaques in both rTg-D HOM and TgSD-AD rats, respectively, with limited to no presence of these markers in WT rats (Figure 11, A and D). Similarly, the robust activation of the TGF-β1 network (Figure 10, A and D) is also supported by the strong expression of TGF-β1 around the periphery of cortical amyloid plaques in both models (Figure 11, E and F), which was previously shown in activated microglia engaged with fibrillar amyloid.^39^ A list of the shared DEPs implicated in the TGF-β1 network is provided in Supplemental Table S6.

## Discussion

Despite the prevalence and significant clinical burden of CAA, the mechanisms underlying the development of severe vasculopathies, such as perivascular inflammation, thrombotic occlusions, vascular degeneration, microhemorrhage, and vascular-mediated cognitive impairment and dementia, resulting from vascular fibrillar Aβ deposition, remain poorly understood. The rTg-D rat was generated to model familial Dutch CAA type-2, providing a more uniform platform for molecular and mechanistic investigation not possible in more heterogeneous human cases.^28^^,^^29^ Because of the relatively slow (18 to 24 months) and somewhat variable nature of pathology development, in the present study, rTg-D rats were bred to homozygosity with the expectation of earlier emergence and more uniform CAA pathology. Indeed, the rTg-D HOM rats develop earlier onset and more robust CAA than rTg-D HEM rats (Table 2, Figure 1, and Supplemental Figure S2), but also presented with abundant fibrillar parenchymal amyloid plaques and dyshoric vascular amyloid (Table 3, Figure 1, and Supplemental Figure S2), driving potent glial responses (Figures 3 and 4), phosphorylated tau pathology (Figure 5), and changes to the cerebral proteome (Figures 6 and 7). Furthermore, the pathology of rTg-D HOM rats is more reminiscent of AD pathology when compared with the TgSD-AD model AD rat model (Figures 8, 9, 10, and 11).

CAA in humans and in rTg-D rats (HEM or HOM) is largely composed of fibrillar Aβ40,^2^^,^^49^ whereas fibrillar parenchymal amyloid plaques in human AD, as well as TgSD-AD rats, are chiefly Aβ42 (Supplemental Figures S3 and S4).^50^, ^51^, ^52^, ^53^ In contrast, the fibrillar parenchymal amyloid plaques that develop in rTg-D HOM rats largely contain Aβ40, like the CAA deposits (Supplemental Figure S2). Interestingly, coarse-grained atypical amyloid plaques observed in early-onset AD tend to be associated with CAA and are composed largely of fibrillar Aβ40,^54^ similar to the fibrillar plaques observed in rTg-D HOM rats. It was recently reported that amyloid fibrils seeded from Dutch-type CAA or sporadic CAA exhibit different structural features.^26^ In future studies, it will be interesting to determine whether amyloid fibrils derived from CAA or fibrillar parenchymal plaques in rTg-D HOM rats exhibit similar or distinct structural features.

rTg-D HEM rats primarily develop fibrillar CAA type-2 in cerebral arterioles and diffuse parenchymal Aβ deposits, whereas fibrillar parenchymal amyloid plaques and phosphorylated tau pathology are largely absent.^28^^,^^29^ This basically reflects the clinical phenotype of patients with familial Dutch-type CAA who are typically hemizygous for the mutant *AβPP* gene and similarly largely lack the presence of fibrillar parenchymal amyloid plaques and phosphorylated tau pathology.^55^ To our knowledge, there have been no reported human cases that are homozygous for familial Dutch-type CAA mutation in the *AβPP* gene. It was somewhat surprising that rTg-D HOM rats developed robust parenchymal amyloid plaques and associated phosphorylated tau. This suggests that the seeding and propagation of parenchymal amyloid plaques may be dependent on Aβ peptide concentration. For example, because the rTg-D HOM rats have twice the copy number of the Dutch mutant human *AβPP* transgenes, they express higher amounts of Dutch mutant human AβPP (Supplemental Figure S1) and produce higher amounts of Dutch mutant Aβ peptides (Table 3). Therefore, the higher amounts of total Dutch mutant Aβ in brain may be what initiates parenchymal plaque formation. Alternatively, although Aβ42 is a minor component of the parenchymal amyloid plaques in rTg-D HOM rats (Supplemental Figure S3), increasing this minor component may be the key driver for seeding fibrillar plaque formation.

Recently, differential proteomic analysis of rTg-D HEM rat brains was reported.^29^ In the present study, few significantly elevated proteins met the predefined threshold of a ≥50% increase. However, many of the previously reported DEPs were identified. There are several potential explanations contributing to the observed differences in these two analyses. First, the original analysis was conducted using an AB Sciex 5600 QTOF mass spectrometer in contrast to the Bruker TimsTOF Pro 2 mass spectrometer used here, with resolution and sensitivity differences resulting in peptide identification and quantification differences. Second, the Spectronaut analysis conducted here was performed with an updated version (version 19.1.240806.62635) referencing a newly developed spectral library that included raw spectral files from a variety of mass spectrometers (as described above) in contrast to the previously used library^36^ developed with raw spectral files from the AB Sciex 5600 QTOF only. Finally, the discovery proteomic analysis here was performed on whole brain tissue, of which the parenchymal tissue accounts for a much greater portion than the cerebral blood vessels. Therefore, DEPs resulting specifically from cerebral vascular pathology in the rTg-D HOM rats, those that would be commonly shared with the rTg-D HEM rats, which display strictly cerebral vascular pathology, comprise only a small portion of the analyzed proteins, with the rTg-D HOM rat DEPs likely dominated by the robust perivascular and parenchymal pathologies. Thus, because of the differences in the models, the proteomics analysis here consequently serves to highlight the differences rather than the similarities between the rTg-D HEM rats and rTg-D HOM rats. Nevertheless, the repeated observed differential increase of APOE, clusterin (CLU), HTRA1, beta-2-microglobulin (B2M), cathepsin D (CTSD), cathepsin S (CTSS), apolipoprotein D (APOD), microtubule-associated protein tau (MAPT), and S100A4 both in previous and present rTg-D HEM rat analyses, as well as in rTg-DI CAA type-1 rat model,^29^^,^^36^, ^37^, ^38^, ^39^, ^40^ further supports the potential of these proteins as biomarkers associated with CAA.

In general, the rTg-D HOM rats displayed much greater similarity in DEPs with the TgSD-AD rats than the rTg-D HEM rats. This is not surprising, as the pathologic features of the rTg-D HOM rats are strikingly similar to those of the TgSD-AD rats. IPA analysis of the DEPs shared between the two models indicated similar activation of phagosome formation and production of nitric oxide and ROS in macrophages (Figure 10). These pathways potentially indicate involvement of peripheral immune cells, such as neutrophils or macrophages, as both phagosome formation and production of ROS contribute to the innate immune responses of these cell types,^43^^,^^44^ although it could also represent similar functions of microglia^45^ and astrocytes.^46^ Notably, the parenchymal amyloid plaque pathology in both rTg-D HOM rats and TgSD-AD rats share robust recruitment and activation of microglia and astrocytes (Figure 8). Additionally, fMLP is a chemoattractant that leads to neutrophil swarming in infection and instances of tissue damage.^47^ The predicted activation of fMLP signaling (Figure 10) further implicates neutrophil swarming, which was supported by the recruitment of neutrophils around parenchymal amyloid plaques in the rTg-D HOM rats and TgSD-AD rats (Figure 11).

It is noteworthy that the TGF-β1 network was also activated in both the rTg-D HOM rats and TgSD-AD rats. DEPs from the TGF-β1 network were previously identified, as well as elevated TGF-β1 levels in rTg-D HEM rats as well as predicted activation and elevation of TGF-β1 levels in the rTg-DI rat model of CAA type-1.^29^^,^^36^^,^^39^ Furthermore, TGF-β1 was elevated in activated microglia engaged with fibrillar amyloid deposits.^56^ Here, elevated TGF-β1 in microglia surrounding parenchymal fibrillar amyloid plaques was observed in both rTg-D HOM rats and TgSD-AD rats (Figure 11). The TGF-β1 network has been implicated in the pathogenesis of both AD and CAA to facilitate the deposition of fibrillar amyloid in cerebral vessels and parenchymal plaques.^57^ Relevant to the present studies, the TGF-β1 network is elevated in Dutch-type CAA human cases.^58^

Interestingly, the serine proteinase HTRA1, a well-reported regulator of TGF-β1 signaling,^59^, ^60^, ^61^, ^62^, ^63^ was consistently elevated across the rTg-D HEM, rTg-D HOM, and TgSD-AD models. HTRA1 is increasingly implicated in CAA with reported increases of HTRA1 in brains of the rat capillary CAA type-1 model rTg-DI,^36^, ^37^, ^38^, ^39^, ^40^ rTg-D HEM rats,^29^ and in human CAA patient isolated cerebral vessels.^64^^,^^65^ Additionally, HTRA1 shows strong colocalization with amyloid deposits in both the rTg-DI and rTg-D HEM models,^29^^,^^36^^,^^39^ and with amyloid deposits in human CAA cases.^64^, ^65^, ^66^ These observations have led to the theory that sequestration of HTRA1 in amyloid deposits reduces its proteolytic activity.^64^^,^^66^

Proteomic evaluation of human CAA and AD has been reported.^64^^,^^65^^,^^67^^,^^68^ Several identified DEPs in the human tissue studies overlap with DEPs observed here in the rTg-D HEM, rTg-D HOM, and TgSD-AD models. For example, CLU, which was found elevated here in rTg-D HOM rats, TgSD-AD rats, and previously in rTg-D HEM rats^29^ and rTg-DI CAA type-1 rats^36^ was similarly identified as elevated in several human tissue studies.^64^^,^^65^^,^^67^ Despite the repeated observation of CLU increases in the rTg-DI, rTg-D HEM, rTg-D HOM, and TgSD-AD rat models, and the agreement of increased CLU in human brain tissue studies, a 20% decrease in CLU in cerebral spinal fluid from patients with CAA was found.^69^ However, CLU directly binds Aβ fibrils and alters their aggregation, and CLU accumulates in CAA affected vessels.^70^^,^^71^ Therefore, the sequestration and accumulation of CLU in Aβ aggregates, resulting in increased CLU in brain tissue, may result in decreased clearance of CLU to the cerebral spinal fluid. In other investigations, Handa et al^68^ reported increased glial fibrillary acidic protein in human CAA cases, which was significantly elevated in the rTg-D HOM rats and TgSD-AD rats, and trending as increased in rTg-D HEM rats, as previously reported,^29^ although here the effect was not statistically significant. These findings are consistent with the extent of astrocyte activation observed in these models. Moreover, Handa et al^68^ reported decreased levels of gamma-synuclein (SNCG), prefoldin subunit 2 (PFDN2), and pyridoxal phosphate binding protein (PLPBP), and increased inorganic pyrophosphatase 1 (PPA1) in human cases, the trajectories that were largely shared by the rTg-D HOM rats and TgSD-AD rats.

Despite the overlap in DEPs observed in the current rat models and those reported in human CAA studies, several potential limitations of these findings need to be considered. Most notably, the species-specific differences between the rat CAA models and human cases may confound translatability of these findings. Additionally, human CAA studies primarily focused on isolated vessels from patients and likely failed to capture surrounding perivascular proteomic changes as well as parenchymal amyloid and tau pathologies present in the rTg-D HOM rats and TgSD-AD rats. Finally, the amyloid deposits observed in the human cases were almost always composed of nonmutated Aβ, whereas Aβ deposits in the rTg-D HEM and HOM models are composed of Aβ40 harboring the familial Dutch E22Q CAA mutation. Considering the unique structure exhibited by Dutch familial mutant Aβ fibrils,^25^ it is possible that they elicit unique cellular and molecular responses, resulting in a unique cerebral proteomic signature compared with nonmutated Aβ fibrils.

Conversely, the use of the rat models for proteomic analyses offers some unique advantages. For example, human cases are by nature heterogeneous and harbor an array of potentially confounding variables, including genetic diversity, and varying environmental, lifestyle, and medical factors that can obscure reliable identification of CAA-induced changes. In contrast, the rat models used in the present study were maintained on a uniform genetic background and housed under controlled environmental and lifestyle conditions, thereby limiting confounders and variance in populations studies. Moreover, the continued use of standard rat models provides the opportunity to evaluate homogeneous populations at designated stages of disease, facilitating the detection of both emergent and chronic alterations in the cerebral proteome, as well as associated changes in relevant biological fluids.

## Disclosure Statement

None declared.

## References

[1] Rensink A.A.M., de Waal R.M.W., Kremer B., Verbeek M.M. (2003). Pathogenesis of cerebral amyloid angiopathy. Brain Res Rev.

[2] Attems J., Jellinger K., Thal D.R., Van Nostrand W. (2011). Review: sporadic cerebral amyloid angiopathy. Neuropathol Appl Neurobiol.

[3] Biffi A., Greenberg S.M. (2011). Cerebral amyloid angiopathy: a systematic review. J Clin Neurol.

[4] Arvanitakis Z., Leurgans S.E., Wang Z., Wilson R.S., Bennett D.A., Schneider J.A. (2011). Cerebral amyloid angiopathy pathology and cognitive domains in older persons. Ann Neurol.

[5] Auriel E., Greenberg S.M. (2012). The pathophysiology and clinical presentation of cerebral amyloid angiopathy. Curr Atheroscler Rep.

[6] Boyle P.A., Yu L., Nag S., Leurgans S., Wilson R.S., Bennett D.A., Schneider J.A. (2015). Cerebral amyloid angiopathy and cognitive outcomes in community-based older persons. Neurology.

[7] Thanvi B., Robinson T. (2006). Sporadic cerebral amyloid angiopathy—an important cause of cerebral haemorrhage in older people. Age Ageing.

[8] Kimberly W.T., Gilson A., Rost N.S., Rosand J., Viswanathan A., Smith E.E., Greenberg S.M. (2009). Silent ischemic infarcts are associated with hemorrhage burden in cerebral amyloid angiopathy. Neurology.

[9] Chung Y.-A., O J.H., Kim J.-Y., Kim K.-J., Ahn K.-J. (2009). Hypoperfusion and ischemia in cerebral amyloid angiopathy documented by 99mTc-ECD brain perfusion SPECT. J Nucl Med.

[10] Okamoto Y., Yamamoto T., Kalaria R.N., Senzaki H., Maki T., Hase Y., Kitamura A., Washida K., Yamada M., Ito H., Tomimoto H., Takahashi R., Ihara M. (2012). Cerebral hypoperfusion accelerates cerebral amyloid angiopathy and promotes cortical microinfarcts. Acta Neuropathol.

[11] Roongpiboonsopit D., Charidimou A., William C.M., Lauer A., Falcone G.J., Martinez-Ramirez S., Biffi A., Ayres A., Vashkevich A., Awosika O.O., Rosand J., Gurol M.E., Silverman S.B., Greenberg S.M., Viswanathan A. (2016). Cortical superficial siderosis predicts early recurrent lobar hemorrhage. Neurology.

[12] Samarasekera N., Smith C., Al-Shahi Salman R. (2012). The association between cerebral amyloid angiopathy and intracerebral haemorrhage: systematic review and meta-analysis. J Neurol Neurosurg Psychiatr.

[13] Boulouis G., Charidimou A., Jessel M.J., Xiong L., Roongpiboonsopit D., Fotiadis P., Pasi M., Ayres A., Merrill M.E., Schwab K.M., Rosand J., Gurol M.E., Greenberg S.M., Viswanathan A. (2017). Small vessel disease burden in cerebral amyloid angiopathy without symptomatic hemorrhage. Neurology.

[14] Greenberg S.M., Gurol M.E., Rosand J., Smith E.E. (2004). Amyloid angiopathy–related vascular cognitive impairment. Stroke.

[15] Thal D.R., Ghebremedhin E., Rüb U., Yamaguchi H., Del Tredici K., Braak H. (2002). Two types of sporadic cerebral amyloid angiopathy. J Neuropathol Exp Neurol.

[16] Rannikmäe K., Kalaria R.N., Greenberg S.M., Chui H.C., Schmitt F.A., Samarasekera N., Al-Shahi Salman R., Sudlow C.L.M. (2014). APOE associations with severe CAA-associated vasculopathic changes: collaborative meta-analysis. J Neurol Neurosurg Psychiatr.

[17] Levy E., Carman M.D., Fernandez-Madrid I.J., Power M.D., Lieberburg I., van Duinen S.G., Bots G.T., Luyendijk W., Frangione B. (1990). Mutation of the Alzheimer's disease amyloid gene in hereditary cerebral hemorrhage, Dutch type. Science.

[18] Van Broeckhoven C., Haan J., Bakker E., Hardy J.A., Van Hul W., Wehnert A., Vegter-Van der Vlis M., Roos R.A. (1990). Amyloid beta protein precursor gene and hereditary cerebral hemorrhage with amyloidosis (Dutch). Science.

[19] Grabowski T.J., Cho H.S., Vonsattel J.P., Rebeck G.W., Greenberg S.M. (2001). Novel amyloid precursor protein mutation in an Iowa family with dementia and severe cerebral amyloid angiopathy. Ann Neurol.

[20] Bugiani O., Giaccone G., Rossi G., Mangieri M., Capobianco R., Morbin M., Mazzoleni G., Cupidi C., Marcon G., Giovagnoli A., Bizzi A., Di Fede G., Puoti G., Carella F., Salmaggi A., Romorini A., Patruno G.M., Magoni M., Padovani A., Tagliavini F. (2010). Hereditary cerebral hemorrhage with amyloidosis associated with the E693K mutation of APP. Arch Neurol.

[21] van Duinen S.G., Castaño E.M., Prelli F., Bots G.T., Luyendijk W., Frangione B. (1987). Hereditary cerebral hemorrhage with amyloidosis in patients of Dutch origin is related to Alzheimer disease. Proc Natl Acad Sci U S A.

[22] Luyendijk W., Bots G.T.A.M., Vegter-van der Vlis M., Went L.N., Frangione B. (1988). Hereditary cerebral haemorrhage caused by cortical amyloid angiopathy. J Neurol Sci.

[23] Wattendorff A.R., Frangione B., Luyendijk W., Bots G.T. (1995). Hereditary cerebral haemorrhage with amyloidosis, Dutch type (HCHWA-D): clinicopathological studies. J Neurol Neurosurg Psychiatr.

[24] Davis J., Van Nostrand W.E. (1996). Enhanced pathologic properties of Dutch-type mutant amyloid beta-protein. Proc Natl Acad Sci U S A.

[25] Melchor J.P., McVoy L., Van Nostrand W.E. (2000). Charge alterations of E22 enhance the pathogenic properties of the amyloid beta-protein. J Neurochem.

[26] Fu Z., Crooks E.J., Irizarry B.A., Zhu X., Chowdhury S., Van Nostrand W.E., Smith S.O. (2024). An electrostatic cluster guides A[beta]40 fibril formation in sporadic and Dutch-type cerebral amyloid angiopathy. J Struct Biol.

[27] Natté R., Maat-Schieman M.L., Haan J., Bornebroek M., Roos R.A., van Duinen S.G. (2001). Dementia in hereditary cerebral hemorrhage with amyloidosis-Dutch type is associated with cerebral amyloid angiopathy but is independent of plaques and neurofibrillary tangles. Ann Neurol.

[28] Davis J., Xu F., Zhu X., Van Nostrand W.E. (2022). rTg-D: a novel transgenic rat model of cerebral amyloid angiopathy type-2. Cereb Circ Cogn Behav.

[29] Schrader J.M., Majchrzak M., Xu F., Lee H., Agostinucci K., Davis J., Benveniste H., Van Nostrand W.E. (2024). Cerebral proteomic changes in the rTg-D rat model of cerebral amyloid angiopathy type-2 with cortical microhemorrhages and cognitive impairments. Neurosci Insights.

[30] Kilkenny C., Browne W.J., Cuthill I.C., Emerson M., Altman D.G. (2010). Improving bioscience research reporting: the ARRIVE guidelines for reporting animal research. PLoS Biol.

[31] Cohen R.M., Rezai-Zadeh K., Weitz T.M., Rentsendorj A., Gate D., Spivak I., Bholat Y., Vasilevko V., Glabe C.G., Breunig J.J., Rakic P., Davtyan H., Agadjanyan M.G., Kepe V., Barrio J.R., Bannykh S., Szekely C.A., Pechnick R.N., Town T. (2013). A transgenic Alzheimer rat with plaques, tau pathology, behavioral impairment, oligomeric a[beta], and frank neuronal loss. J Neurosci.

[32] Koundal S., Chen X., Gursky Z., Lee H., Xu K., Liang F., Xie Z., Xu F., Lin H.-M., Van Nostrand W.E., Gu X., Elkin R., Tannenbaum A., Benveniste H. (2024). Divergent brain solute clearance in rat models of cerebral amyloid angiopathy and Alzheimer's disease. iScience.

[33] Van Nostrand W.E., Wagner S.L., Suzuki M., Choi B.H., Farrow J.S., Geddes J.W., Cotman C.W., Cunningham D.D. (1989). Protease nexin-II, a potent antichymotrypsin, shows identity to amyloid beta-protein precursor. Nature.

[34] DeMattos R.B., O’dell M.A., Parsadanian M., Taylor J.W., Harmony J.A.K., Bales K.R., Paul S.M., Aronow B.J., Holtzman D.M. (2002). Clusterin promotes amyloid plaque formation and is critical for neuritic toxicity in a mouse model of Alzheimer's disease. Proc Natl Acad Sci U S A.

[35] Johnson-Wood K., Lee M., Motter R., Hu K., Gordon G., Barbour R., Khan K., Gordon M., Tan H., Games D., Lieberburg I., Schenk D., Seubert P., McConlogue L. (1997). Amyloid precursor protein processing and A[beta]42 deposition in a transgenic mouse model of Alzheimer disease. Proc Natl Acad Sci U S A.

[36] Schrader J.M., Xu F., Van Nostrand W.E. (2021). Distinct brain regional proteome changes in the rTg-DI rat model of cerebral amyloid angiopathy. J Neurochem.

[37] Schrader J.M., Xu F., Lee H., Barlock B., Benveniste H., Van Nostrand W.E. (2022). Emergent white matter degeneration in the rTg-DI rat model of cerebral amyloid angiopathy exhibits unique proteomic changes. Am J Pathol.

[38] Schrader J.M., Stanisavljevic A., Xu F., Van Nostrand W.E. (2022). Distinct brain proteomic signatures in cerebral small vessel disease rat models of hypertension and cerebral amyloid angiopathy. J Neuropathol Exp Neurol.

[39] Schrader J.M., Xu F., Agostinucci K.J., DaSilva N.A., Van Nostrand W.E. (2024). Longitudinal markers of cerebral amyloid angiopathy and related inflammation in rTg-DI rats. Sci Rep.

[40] Stanisavljevic A., Schrader J.M., Zhu X., Mattar J.M., Hanks A., Xu F., Majchrzak M., Robinson J.K., Van Nostrand W.E. (2022). Impact of non-pharmacological chronic hypertension on a transgenic rat model of cerebral amyloid angiopathy. Front Neurosci.

[41] Wiśniewski J.R., Rakus D. (2014). Multi-enzyme digestion FASP and the “total protein approach”-based absolute quantification of the Escherichia coli proteome. J Proteomics.

[42] Krämer A., Green J., Pollard J., Tugendreich S. (2014). Causal analysis approaches in Ingenuity Pathway Analysis. Bioinformatics.

[43] Lee H.-J., Woo Y., Hahn T.-W., Jung Y.M., Jung Y.-J. (2020). Formation and maturation of the phagosome: a key mechanism in innate immunity against intracellular bacterial infection. Microorganisms.

[44] Manoharan R.R., Prasad A., Pospíšil P., Kzhyshkowska J. (2024). ROS signaling in innate immunity via oxidative protein modifications. Front Immunol.

[45] Smith A.N., Shaughness M., Collier S., Hopkins D., Byrnes K.R. (2022). Therapeutic targeting of microglia mediated oxidative stress after neurotrauma. Front Med (Lausanne).

[46] Morizawa Y.M., Hirayama Y., Ohno N., Shibata S., Shigetomi E., Sui Y., Nabekura J., Sato K., Okajima F., Takebayashi H., Okano H., Koizumi S. (2017). Reactive astrocytes function as phagocytes after brain ischemia via ABCA1-mediated pathway. Nat Commun.

[47] Lämmermann T. (2016). In the eye of the neutrophil swarm—navigation signals that bring neutrophils together in inflamed and infected tissues. J Leukoc Biol.

[48] Shin M.K., Jang Y.H., Yoo H.J., Kang D.W., Park M.H., Kim M.K., Song J.H., Kim S.D., Min G., You H.K., Choi K.-Y., Bae Y.-S., Min D.S. (2011). N-formyl-methionyl-leucyl-phenylalanine (fMLP) promotes osteoblast differentiation via the N-formyl peptide receptor 1-mediated signaling pathway in human mesenchymal stem cells from bone marrow. J Biol Chem.

[49] Attems J., Jellinger K.A. (2004). Only cerebral capillary amyloid angiopathy correlates with Alzheimer pathology—a pilot study. Acta Neuropathol.

[50] Fang X., Border J.J., Rivers P.L., Zhang H., Williams J.M., Fan F., Roman R.J. (2023). Amyloid beta accumulation in TgF344-AD rats is associated with reduced cerebral capillary endothelial Kir2.1 expression and neurovascular uncoupling. Geroscience.

[51] Borchelt D.R., Thinakaran G., Eckman C.B., Lee M.K., Davenport F., Ratovitsky T., Prada C.-M., Kim G., Seekins S., Yager D., Slunt H.H., Wang R., Seeger M., Levey A.I., Gandy S.E., Copeland N.G., Jenkins N.A., Price D.L., Younkin S.G., Sisodia S.S. (1996). Familial Alzheimer's disease–linked presenilin 1 variants elevate A[beta]1–42/1–40 ratio in vitro and in vivo. Neuron.

[52] Kakuda N., Miyasaka T., Iwasaki N., Nirasawa T., Wada-Kakuda S., Takahashi-Fujigasaki J., Murayama S., Ihara Y., Ikegawa M. (2017). Distinct deposition of amyloid-[beta] species in brains with Alzheimer's disease pathology visualized with MALDI imaging mass spectrometry. Acta Neuropathol Commun.

[53] Iwatsubo T., Odaka A., Suzuki N., Mizusawa H., Nukina N., Ihara Y. (1994). Visualization of A[beta]42(43) and A[beta]40 in senile plaques with end-specific A[beta] monoclonals: evidence that an initially deposited species is A[beta]42(43). Neuron.

[54] Boon B.D.C., Bulk M., Jonker A.J., Morrema T.H.J., van den Berg E., Popovic M., Walter J., Kumar S., van der Lee S.J., Holstege H., Zhu X., Van Nostrand W.E., Natté R., van der Weerd L., Bouwman F.H., van de Berg W.D.J., Rozemuller A.J.M., Hoozemans J.J.M. (2020). The coarse-grained plaque: a divergent A[beta] plaque-type in early-onset Alzheimer's disease. Acta Neuropathol.

[55] Maat-Schieman M., Roos R., Van Duinen S. (2005). Hereditary cerebral hemorrhage with amyloidosis-Dutch type. Neuropathology.

[56] Zhu X., Schrader J.M., Irizarry B.A., Smith S.O., Van Nostrand W.E. (2022). Impact of A[beta]40 and A[beta]42 fibrils on the transcriptome of primary astrocytes and microglia. Biomedicines.

[57] Moursel L.G., Munting L.P., van der Graaf L.M., van Duinen S.G., Goumans M.-J.T.H., Ueberham U., Natté R., van Buchem M.A., van Roon-Mom W.M.C., van der Weerd L. (2018). TGF[beta] pathway deregulation and abnormal phospho-SMAD2/3 staining in hereditary cerebral hemorrhage with amyloidosis-Dutch type. Brain Pathol.

[58] Grand Moursel L., van Roon-Mom W.M.C., Kiełbasa S.M., Mei H., Buermans H.P.J., van der Graaf L.M., Hettne K.M., de Meijer E.J., van Duinen S.G., Laros J.F.J., van Buchem M.A., ’t Hoen P.A.C., van der Maarel S.M., van der Weerd L. (2018). Brain transcriptomic analysis of hereditary cerebral hemorrhage with amyloidosis-Dutch type. Front Aging Neurosci.

[59] Shiga A., Nozaki H., Yokoseki A., Nihonmatsu M., Kawata H., Kato T., Koyama A., Arima K., Ikeda M., Katada S., Toyoshima Y., Takahashi H., Tanaka A., Nakano I., Ikeuchi T., Nishizawa M., Onodera O. (2011). Cerebral small-vessel disease protein HTRA1 controls the amount of TGF-[beta]1 via cleavage of proTGF-[beta]1. Hum Mol Genet.

[60] Oka C., Tsujimoto R., Kajikawa M., Koshiba-Takeuchi K., Ina J., Yano M., Tsuchiya A., Ueta Y., Soma A., Kanda H., Matsumoto M., Kawaichi M. (2004). HtrA1 serine protease inhibits signaling mediated by Tgf[beta] family proteins. Development.

[61] Launay S., Maubert E., Lebeurrier N., Tennstaedt A., Campioni M., Docagne F., Gabriel C., Dauphinot L., Potier M.C., Ehrmann M., Baldi A., Vivien D. (2008). HtrA1-dependent proteolysis of TGF-[beta] controls both neuronal maturation and developmental survival. Cell Death Differ.

[62] Beaufort N., Scharrer E., Kremmer E., Lux V., Ehrmann M., Huber R., Houlden H., Werring D., Haffner C., Dichgans M. (2014). Cerebral small vessel disease-related protease HtrA1 processes latent TGF-[beta] binding protein 1 and facilitates TGF-[beta] signaling. Proc Natl Acad Sci U S A.

[63] Graham J.R., Chamberland A., Lin Q., Li X.J., Dai D., Zeng W., Ryan M.S., Rivera-Bermúdez M.A., Flannery C.R., Yang Z. (2013). Serine protease HTRA1 antagonizes transforming growth factor-[beta] signaling by cleaving its receptors and loss of HTRA1 in vivo enhances bone formation. PLoS One.

[64] Zellner A., Müller S.A., Lindner B., Beaufort N., Rozemuller A.J.M., Arzberger T., Gassen N.C., Lichtenthaler S.F., Kuster B., Haffner C., Dichgans M. (2022). Proteomic profiling in cerebral amyloid angiopathy reveals an overlap with CADASIL highlighting accumulation of HTRA1 and its substrates. Acta Neuropathol Commun.

[65] Hondius D.C., Eigenhuis K.N., Morrema T.H.J., van der Schors R.C., van Nierop P., Bugiani M., Li K.W., Hoozemans J.J.M., Smit A.B., Rozemuller A.J.M. (2018). Proteomics analysis identifies new markers associated with capillary cerebral amyloid angiopathy in Alzheimer's disease. Acta Neuropathol Commun.

[66] Young K.Z., Xu G., Keep S.G., Borjigin J., Wang M.M. (2021). Overlapping protein accumulation profiles of CADASIL and CAA. Am J Pathol.

[67] Manousopoulou A., Gatherer M., Smith C., Nicoll J.A.R., Woelk C.H., Johnson M., Kalaria R., Attems J., Garbis S.D., Carare R.O. (2017). Systems proteomic analysis reveals that clusterin and tissue inhibitor of metalloproteinases 3 increase in leptomeningeal arteries affected by cerebral amyloid angiopathy. Neuropathol Appl Neurobiol.

[68] Handa T., Sasaki H., Takao M., Tano M., Uchida Y. (2022). Proteomics-based investigation of cerebrovascular molecular mechanisms in cerebral amyloid angiopathy by the FFPE-LMD-PCT-SWATH method. Fluids Barriers CNS.

[69] Vervuurt M., Schrader J.M., de Kort A.M., Kersten I., Wessels H.J.C.T., Klijn C.J.M., Schreuder F.H.B.M., Kuiperij H.B., Gloerich J., Van Nostrand W.E., Verbeek M.M. (2024). Cerebrospinal fluid shotgun proteomics identifies distinct proteomic patterns in cerebral amyloid angiopathy rodent models and human patients. Acta Neuropathol Commun.

[70] Kim Y.-M., Park S., Choi S.Y., Oh S.B., Jung M., Pack C.-G., Hwang J.J., Tak E., Lee J.-Y. (2022). Clusterin binding modulates the aggregation and neurotoxicity of amyloid-[beta](1-42). Mol Neurobiol.

[71] Endo Y., Hasegawa K., Nomura R., Arishima H., Kikuta K., Yamashita T., Inoue Y., Ueda M., Ando Y., Wilson M.R., Hamano T., Nakamoto Y., Naiki H. (2019). Apolipoprotein E and clusterin inhibit the early phase of amyloid-[beta] aggregation in an in vitro model of cerebral amyloid angiopathy. Acta Neuropathol Commun.

